# Competing societal objectives in epidemic mitigation: a modeling study of COVID-19 in the Philippines

**DOI:** 10.3389/fpubh.2025.1662043

**Published:** 2025-11-03

**Authors:** Rob Johnson, Rolly Czar Joseph T. Castillo, Cymon Lubangco, Timothy Robin Teng, Mark Tolentino, Christian Morgenstern, Patrick Doohan, David Haw, Giovanni Forchini, Elvira P. de Lara-Tuprio, Joselito T. Sescon, Katharina Hauck

**Affiliations:** ^1^MRC Centre for Global Infectious Disease Analysis & WHO Collaborating Centre for Infectious Disease Modelling, Jameel Institute for Disease and Emergency Analytics, Imperial College London, London, United Kingdom; ^2^Department of Economics, Ateneo de Manila University, Quezon City, Philippines; ^3^BSP Research Academy, Bangko Sentral ng Pilipinas, Manila, Philippines; ^4^Department of Mathematics, Ateneo de Manila University, Quezon City, Philippines; ^5^Department of Mathematical Sciences/Institute of Veterinary, Infection and Ecological Sciences, University of Liverpool, Liverpool, United Kingdom; ^6^Umeå School of Business, Economics and Statistics, Umeå Universitet, Umeå, Sweden

**Keywords:** epidemiology, infectious disease, COVID-19, economics, poverty, pandemic preparedness

## Abstract

School closures and suspension of non-essential economic activities are highly effective respiratory-pandemic mitigation strategies because they effectively interrupt disease transmission. However, they come with high societal costs. Most of these costs are borne by workers who lose their income, especially those who are not supported by welfare benefits, and students whose future income depends on their education. In countries where many households live close to the poverty line, closures should be designed to minimize impacts on the most vulnerable. The objective of this study is to learn and compare policy responses that minimize the number of people that fall below the poverty line, maximize GDP, or maximize societal welfare in a model of the COVID-19 outbreak in the Philippines. Toward this objective, we quantify societal welfare in terms of lives, education, GDP, and we introduce poverty as a novel fourth dimension. We then use a population microsimulation model , an epidemiological model , and GDP and education projections to determine intervention strategies involving the partial closure of schools and economic sectors with the objective of mitigating the epidemic while minimizing societal losses. We find the cost of reducing poverty is substantial in terms of the other outcomes, making a case for poverty reduction as an important tool for increasing societal resilience and preparedness for crises such as pandemics. From a modeling perspective, we identify the need for timely data collection in order to create tools to assist in future epidemics.

## 1 Introduction

Governments around the world responded to the COVID-19 pandemic with mandates to reduce in-person interactions and therefore the spread of infection in the population. Mandates, including stay-at-home orders, business closures and school closures, restricted economic activities. Restrictions affected the demand side of the economy by reducing consumption of goods and services requiring in-person contact, and the supply side via reductions in labor supply. Adverse impacts were mitigated to some extent through increased online consumption, teleworking and online schooling.

Restricting activities not essential to daily life constitutes effective pandemic mitigation by reducing opportunities for community transmission. These restrictions often come in the form of economic and school closures. However, there is a high societal cost associated with the imposition of such closures. This damage was mitigated in some high-income countries using extensive welfare programmes (1, 2). The same cannot be said, however, for low- and middle-income countries which have lower social welfare budgets and less recourse to government financing instruments. Economic closures particularly impacted those informally employed or with low income, and widened income and wealth inequalities (3–5). School closures, on the other hand, interrupted education which can diminish lifelong earnings and, consequently, the nation's economic growth (6, 7).

The purpose of this study is to compare closure strategies in terms of deaths, education lost, GDP lost, and the number of households that fall below the line of poverty. We use an existing model previously applied to Indonesia (8), augmented with a microsimulation model of household incomes, to project the health, economic, poverty and educational outcomes. We apply the model to the first wave of COVID-19 in the Philippines. We compare the outcomes of closure strategies that maximize GDP with those that minimize poverty. This allows us to quantify the magnitude of the trade-off between competing societal objectives when employing closures; for example, what it might cost in terms of aggregate economic output or months of schooling to prevent vulnerable households from falling below the poverty line. Including poverty in societal cost is an important extension to existing models, making them more relevant for low- and middle-income countries.

## 2 Background

### 2.1 Impact of the COVID-19 pandemic on poverty and education

The COVID-19 pandemic increased poverty globally, and particularly in low- and middle-income countries. The World Bank estimated that, in 2021, approximately 97 million more people were living in extreme poverty as a result of the pandemic, and around 163 million more people lived on less than $5.50 per day, raising the global poverty rate from 7.8% to 9.1% (9). Meanwhile, UNICEF estimated that up to 86 million children fell into poverty in 2020, representing a 15% increase (10).

These patterns were seen also in the Philippines: The incidence of poverty in the Philippines decreased from 23.5% to 16.7% between 2015 and 2018, but then increased again to 18.1% in 2021. Incidence of food poverty decreased from 9.1% to 5.2%, and then increased again to 5.9% (11).

School closures were employed extensively worldwide (12), for longer than a year in many LMICs, including the Philippines (13). Education losses were high, and increased with prolongation of closures (14) despite rapid deployment of remote teaching programmes (15). The ultimate cost of these learning losses to societies is difficult to anticipate (16, 17). It has been estimated that 12 weeks of school closures will reduce productivity 45 years later by 0.4% (7). There are other societal and economic costs of school closures, such as detrimental impacts on the social and personal development of children, parents not being able to work, and reduced demand for goods and services from suppliers to education sectors (18).

The Philippines was one of the last countries in the world to re-open schools (19). It was estimated that the first year of school closures, in which remote teaching was estimated to be 37% as effective as in-person teaching, would cost the economy 10.7 trillion PHP over the next 40 years, equivalent to 60% of the country's GDP in 2020. Concurrently, school closures would result in an estimated GDP loss of 230 billion PHP in 2020 due to lost economic activity (20). However, while closures impacted enrollment in other places (21), there was no long-term (or persistent) drop out of students in the Philippines. Enrollment for the academic year 2020–21 was 96.5% of its value in 2019–20, and in 2021–22 it was 104% of its value in 2020–21 (22).

The preceding discussion demonstrates the severe impact of the COVID-19 pandemic on education and poverty in many countries across the globe. This is further exacerbated by restrictive policies that primarily focused on mitigating the impacts on public health, particularly on the number of infections and deaths.

### 2.2 Pandemic mitigation

With the threat of severe future pandemics potentially growing (23), and a wealth of data collated from all over the world on the impacts of the COVID-19 pandemic and its mitigation, now is the time to develop new models that can assist policymakers in navigating future crises. Some of the costs associated with the pandemic, such as lost education, were a direct consequence of pandemic mitigation policies. Balancing these competing priorities is the focus of this work.

In high-income countries, adverse impacts on the economy and households' livelihoods were mitigated with fiscal relief in the form of subsidies to businesses and individuals. Large-scale welfare programs were not a viable option for many LMICs. The IMF estimated that, by October 2021, the UK had spent about 19.3% of their GDP on discretionary fiscal response to the COVID-19 crisis, while the equivalent value for the Philippines was 4.45% (24). This includes the Social Amelioration Program, through which 10,000 to 16,000 PHP per household was distributed to up to 18 million households by November 2020, totalling 182.9 billion PHP (0.9% of GDP) (25).

For an LMIC with limited resources to implement welfare programs, a key objective for pandemic mitigation, in addition to averting deaths, is minimizing harm to the most vulnerable due to income loss. Implementing closures that reduce the number of deaths might come at the cost of households falling below the threshold of poverty. Conversely, protecting livelihoods may come at the cost of a higher number of deaths, or of closing schools in order to reduce transmission. Prioritizing livelihoods over aggregate GDP might entail keeping low-income workers in their jobs at the expense of higher-income workers. In summary, policy makers need to navigate a complex trade-off between four components of societal welfare: health, GDP, poverty, and education.

Epidemiological models have been used worldwide to monitor the public health impact of the COVID-19 epidemic and to project impacts of public health interventions (26, 27). Similarly, economic modeling has been used to project economic impacts of the pandemic, and inform the design of fiscal and monetary relief and recovery policies. However, there was often little connection between the policies analyzed by epidemiologists and economists, which made it difficult for decision makers to assess projected impacts of policies across multiple societal objectives. There potentially are feedback effects between epidemiology and the economy that cannot be captured by independent analyses. There were also few models that could provide concrete insights, and therefore actionable recommendations, on overall societal impacts of the pandemic and its mitigation across health, economic, and social outcomes (28–30). This is the methodological gap we propose to address.

## 3 Methods

We start by adapting an existing model to project the impact of pandemic mitigation strategies on societal welfare. We then define a social welfare function (SWF) with four components: pandemic deaths, educational loss, aggregate income, and poverty. By codifying different societal objectives within one objective function, we seek socially “optimal” pandemic mitigation policies.

### 3.1 The model

**Figure 1:**
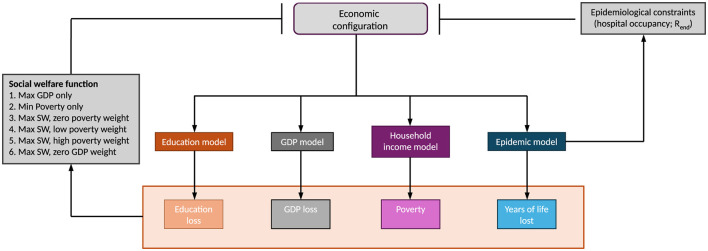
Graphical illustration of the method. Arrows represent model inputs and outcomes. Bars represent optimisation constraints. The economic configuration determines the extent of education loss via school closures, the GDP loss, the number of households that fall below the line of poverty, and the years of life lost via independent models. We are choosing an economic configuration of sector closures to minimize social welfare loss subject to epidemiological constraints, which come from the epidemic model. The constraints are that hospital occupancy never breaches available capacity, and *R*_end_ at the end of projection does not exceed 1. We optimize six alternative objective functions. Two place different weights on GDP and poverty as maximand/minimand. Objective functions 3 to 6 include years of life lost as part of the social welfare function, whereas objective functions 1 and 2 do not.

Our starting point is the DAEDALUS model of Haw et al. (29) , which we augment with a model estimating the impact of mandated closures on poverty. With this joint model, we simulate the first six months of the COVID-19 pandemic in the Philippines, and seek the closure policy that minimizes societal losses. The method is shown graphically in Figure 1.

We use a 34-sector representation of the Philippine economy following the classification of UN Economic and Social Affairs (31), whose features are described in Supplementary material C.2. A closure policy, or “economic configuration”, is a numerical representation of how closed each of the 34 economic sectors is in each month over the period in question, expressed as the % reduction in gross value added (GVA) compared to 2019. The economic configuration determines GDP (as aggregate GVA) and poverty via reductions in production and household income.

The economic configuration also determines how open schools are via the education sector, and the extent of transmission in the epidemiological model, which ultimately determines the number of deaths. Closures manifest as a reduction in the number of contacts made between individuals. Closures imply that worker-to-worker, worker-to-customer and customer-to-customer contacts are reduced. We estimate hospital occupancy and the number of deaths for a given economic configuration using DAEDALUS. DAEDALUS is a compartmental epidemic model stratified by age and sector of employment that takes into account the economic configuration via sector-specific contact rates. The model is explained in Supplementary material E. We assume the extent of transmission over the projection horizon to be equal to that estimated for the month of March 2020 using the DAEDALUS model, which was calibrated to hospitalization data (Supplementary material F).

The objective is to find the economic configuration that maximizes social welfare subject to the epidemiological constraints: that hospital capacity is never exceeded; and that, at the end of the period, the effective reproduction number *R*_end_ is no more than 1 and hospital occupancy no more than 50% of maximum hospital capacity. We also specify that the agricultural sector remains open at all times.

### 3.2 Social welfare function

We construct a SWF in terms of gross domestic product (GDP), lives lost, education delivered, and person–months spent below the line of poverty. The SWF is defined as


(1) $$\begin{array}{l} \text{SW}=w_{e}E+w_{g}G-w_{p}P-w_{l}L, \end{array}$$

where SW is social welfare, *E* is the total effective months of education delivered, *G* is the total GDP for the period, *P* is the total number of person–months spent below the line of poverty, and *L* is the number of discounted life years lost (Supplementary material B.2). They each have an associated weight, *w*, that represents their monetary valuation for computing social welfare. In Sections 3.2.1 to 3.2.4 we describe how we compute these quantities.

We define and evaluate six objective functions (Table 1). Each represents possible societal preferences to guide pandemic mitigation and is defined by the choice of *w* values in Equation 1. Objective 1 (Max GDP) is to maximize GDP subject to the epidemiological constraints, as in Haw et al. (29); the education, poverty and life valuations (Figure 1) are all 0. Objective 2 (Min poverty) is to minimize poverty, the number of person–months spent below the line of poverty due to mandated closures (with no contribution from any other component). Objective 3 (Max SW, zero poverty weight) is to maximize social welfare with three components: education, GDP and discounted life years, with a valuation of 1.9 trillion PHP for a month of education lost, and a valuation of 2.4 million PHP for a lost discounted life year. Objective 4 (Max SW, low poverty weight) includes poverty as a fourth component, with a weight of 10,000 PHP per person–month spent below the poverty line. Objective 5 (Max SW, high poverty weight) uses a higher poverty valuation of 82,000 PHP per person–month. Objective 6 (Max SW, zero GDP weight) includes education and discounted life-year weights as before, the lower valuation for poverty, and zero weight for GDP.

**Table 1:** Objective function definitions for pandemic mitigation strategies.

**Objective**	**Objective shorthand**	***w*_*e*_**	***w*_*g*_**	***w*_*p*_**	***w*_*l*_**
1	Max GDP	0	1	0	0
2	Min poverty	0	0	1	0
3	Max SW, zero poverty weight	1.9 trillion	1	0	2.4 million
4	Max SW, low poverty weight	1.9 trillion	1	10,000	2.4 million
5	Max SW, high poverty weight	1.9 trillion	1	82,000	2.4 million
6	Max SW, zero GDP weight	1.9 trillion	0	10,000	2.4 million

The weights reflect valuations for components of social welfare. Values for discounted life years and education make use of published estimates.

#### 3.2.1 Life years lost due to COVID-19 deaths

Lives lost are converted into monetary terms using a value of a statistical life (VSL) approach (32). We rely on the intrinsic rather than instrumental interpretation of the VSL (33), which is based on individuals' willingness to pay to reduce their own risk of mortality.

For each simulation, we estimate the total number of discounted years of life lost by multiplying the number of deaths per age group by their (discounted) expected years of life remaining. The years lost are then valued with the value of a discounted statistical life year (VdLY), which we derive from the VSL (Supplementary material B.2). We interpret the VSL as a population-weighted average following Ananthapavan et al. (34) and Robinson et al. (35), where each age group has a VSL defined by the number of expected life years remaining, and where each discounted life year has the same value.

We use an estimate of the VSL for the Philippines from Palanca-Tan (36), assuming the study applies to people aged 0 to 14, and that the VSL is constant with respect to average income. This gives us a VdLY estimate for 2020 of 2,400,000 PHP, which is approximately 50,000 USD.

The VSL estimate from Palanca-Tan (36) is similar to estimates derived from two other sources. Following Viscusi and Masterman (37), assuming a linear relationship between VSL and GDP, and an average of 7% growth from 2015, we estimate a VdLY for 2020 of 1,700,000 PHP. Following Saluja et al. (38), where the VSL with population-weighted means by World Health Organization region and World Bank income group is reported for Western Pacific countries, we estimate a VdLY of 2,000,000 PHP.

For the objectives that include lives lost, we set *w*_*l*_ = VdLY in Equation 1. For the other objectives, we set *w*_*l*_ = 0.

#### 3.2.2 Education lost due to school closures

The value of a school year is the value that one year of education adds to a person's income over their lifetime. To estimate the societal loss from school closures, we estimate the total effective education missed due to closures, and the impact it has on pupils' life-long income.

Education is quantified as the effective number of months of schooling. In each month τ = 1, ..., *T* of the projection period, education is divided into a fraction of in-person teaching (*x*_ed, τ_) and remote teaching (1−*x*_ed, τ_). We compute the total amount of education completed as a weighted sum of school openness and its complement, where remote teaching contributes a fraction *s* of the educational value of in-person teaching:


$$E=\overset{T}{\underset{\tau =1}{\sum }}\left(x_{\text{ed},\tau }+s\left(1-x_{\text{ed},\tau }\right)\right).$$

For the effectiveness of remote teaching, we use an extant estimate of *s* = 0.37 (20).

One year of remote teaching (at 37% effectiveness) was estimated to cost the economy 10.7 trillion PHP over the next 40 years, which equates 89% of the country's GDP in 2019 to one full year lost (20). This aligns with an estimate from Psacharopoulos et al. (39), where a whole year of lost education costs 73% of GDP for middle-income countries, assuming that closures affect 90% of students.

Using the estimate of 10.7 trillion PHP, and that there are nine months of teaching in the academic year, of which four (April, May, June, September) fall in the projection horizon, the value of a month of education is 1.9 trillion PHP, which is 82,000 PHP per person per month. Thus we use *w*_*e*_= 1.9 trillion for the SWF where education is to be valued, and *w*_*e*_ = 0 for the other objectives.

#### 3.2.3 GDP lost due to business closures

GDP is naturally measured in monetary terms. GDP over the period is the sum over all *S*−1 sectors (we omit the education sector as we estimate the loss due to school closures separately) and all *T* months of monthly GVA per sector, scaled by the extent to which the sector is open according to the mandated economic configuration. Writing sector openness as *x*_*s*, τ_ for sector *s* in month τ, the GDP can be expressed as


$$\text{GDP}=\overset{S-1}{\underset{s=1}{\sum }}\overset{T}{\underset{\tau =1}{\sum }}x_{s,\tau }{\text{GVA}}_{s}.$$

We approximate a sector's monthly GVA as annual GVA from 2019 divided by 12.

#### 3.2.4 Poverty due to business closures

To model poverty, we estimate the expected number of people living below the line of poverty as a function of the households' workers' incomes, their probabilities to retain their income given sector closure, and the extent to which sectors are closed. Summing over the income of all workers within households and adding non-work income, we identify which combinations of workers are sufficient for the household to remain above the line of poverty. We write the probability that the household remains above the line as a function of the workers' probabilities to lose their income given their sectors' closures.

By definition, the poverty threshold is applied to household income per capita, a measure that distributes total household income across all members of the household irrespective of what they contribute to total household income. This means that either all members of a household, or none of its members, are below the threshold. To project excess poverty requires us to calculate household income per capita for each household. Following Vos and Sánchez (40), we write household income per capita in a month as


$$ypc_{h}=\frac{1}{n_{h}}\left[\overset{n_{h}}{\underset{i=1}{\sum }}yp_{hi}+yq_{h}\right],$$

where *n*_*h*_ is the number of people in the household, *yp*_*hi*_ is the (estimated) income of person *i* in household *h*, and *yq*_*h*_ is non-work income for the household obtained from the family income and expenditure survey [FIES, (41)]. We assume that non-work income *yq*_*h*_ is unaffected by closures.

To estimate *yp*_*hi*_, we use two data sources. The first is FIES, which reports total annual income from formal and informal employment, non-work-related income, and the main sector of employment of one member of the household. The second is the labor force survey [LFS, (42)], which reports data for all workers in each household covered by FIES on main sector of employment, usual number of hours worked, number of hours worked in the past week, and the basis of pay (whether wage or entrepreneurial). For those in waged employment, the usual daily pay is reported. Income data is missing for entrepreneurial workers. We impute the hourly rate of pay by matching individual workers to their nearest neighbors and sampling with replacement (package knn in R; see Supplementary Figure S1). We then scale the rate based on the difference seen in hourly income between waged and entrepreneurial income for households with income data in the FIES, using only the subset of households where there is exactly one worker and one type of work income in the household (see Supplementary Figure S2). See Supplementary material C1 for details of the surveys.

Income *yp*_*hi*_ counts toward household income depending on the openness of the sector of work of person *i* in household *h* in month τ and on the individual's personal probability to lose their income relative to others in their sector. The openness of sector *s* in month τ is given as *x*_*s*, τ_, and the sector that individual *i* of household *h* works in is given as *S*_*hi*_. We make the simplifying assumption that closure of a sector to, say, 70% means that 30% people lose their income and everyone else's remains unaffected.

We represent the population using a microsimulation model, where *ypc*_*h*, τ, *j*_ is the income per capita of household *h* in month τ in sample *j*, which depends on sector closure in month τ. We compute it as


(2) $$\begin{array}{l} ypc_{h,\tau ,j}=\frac{1}{n_{h}}\left[\overset{n_{h}}{\underset{i=1}{\sum }}p_{hi,\tau ,j}yp_{hi}+yq_{h}\right]. \end{array}$$

with


$$p_{hi,\tau ,j}\sim \text{Bernoulli}\left({\hat{p}}_{hi,\tau }\right),$$

and


(3) $$\begin{array}{l} {\hat{p}}_{hi,\tau }=\{\begin{array}{ll} p_{hi}^{\left(0\right)}x_{S_{hi},\tau }/{\bar{p}}_{S_{hi}} & x_{S_{hi},\tau }<{\bar{p}}_{S_{hi}}\\ 1-\left(1-p_{hi}^{\left(0\right)}\right)\left(1-x_{S_{hi},\tau }\right)/\left(1-{\bar{p}}_{S_{hi}}\right) & x_{S_{hi},\tau }>{\bar{p}}_{S_{hi}} \end{array} \end{array}$$

Here, *S*_*hi*_ represents the sector of the *i*th person in household *h*. $p_{hi}^{\left(0\right)}$ is the probability of person *hi* to keep their job based on a logistic regression, using predicted income and demographic variables such as level of education, sex, age and region as independent variables.

Across all individuals in the sector, the mean probability of job retention is ${\bar{p}}_{S_{hi}}$. Equation 3 computes scaled values ${\hat{p}}_{hi,\tau }$ so that all probabilities in the sector yield a mean equal to *x*_*S*_*hi*_, τ_, the sector openness.

We write the total number of person–months spent below the line of poverty over *T* months based on *H* households in simulated sample *j* as


$$P_{j}=\overset{T}{\underset{\tau =1}{\sum }}\overset{H}{\underset{h=1}{\sum }}w_{h}\cdot n_{h}\cdot 1_{h,\tau ,j},$$

where *w*_*h*_ is the survey weight of household *h*, and


(4) $$\begin{array}{l} 1_{h,\tau ,j}=\{\begin{array}{ll} 1 & ypc_{h,\tau ,j}<ypc^{*}\\ 0 & \text{ otherwise.} \end{array} \end{array}$$

The monthly threshold is ypc^*^ = 2152.08, which we obtain by dividing the annual threshold for the Philippines in 2018 [25,813 PHP (11)] by 12.

A microsimulation model requires thousands of iterations over index *j* to evaluate its expectation. Because we are employing an optimisation routine, which itself is computationally demanding, we cannot embed the microsimulation model as it is within the framework. Instead, we pre-determine the probability that **1**_*h*, τ, *j*_ = 0 (Equation 4) as a function of workers by identifying which combination(s) of workers in the household are sufficient to keep the household above the line of poverty. This yields one expression per household, $f_{h}\left(\left\{p_{h}^{\left(0\right)}\right\},x_{\tau }\right)$, representing the probability that this condition is met in terms of sector closures, *x*_τ_, and each worker's relative probability to lose their income, $\left\{p_{h}^{\left(0\right)}\right\}$, via Equation 3 (see Supplementary Figure S3). This is easily represented by Venn diagrams for households with only two or three workers (Supplementary Figure S4). Each household contributes a number of people *n*_*h*_ multiplied by the household weight *w*_*h*_ to the count below the poverty threshold with the household-specific function:


(5) $$\begin{array}{l} P=\overset{T}{\underset{\tau =1}{\sum }}\overset{H}{\underset{h=1}{\sum }}w_{h}\cdot n_{h}\left(1-f_{h}\left(\left\{p_{h}^{\left(0\right)}\right\},x_{\tau }\right)\right). \end{array}$$

In addition to excess poverty (the person–months that are spent in poverty due to pandemic mitigation), we also report total poverty over the projection horizon (pre-existing plus excess poverty). Being below the threshold is a binary measure that does not inform on the extent of poverty. To this end, we report the poverty shortfall. This is the amount that it would cost to bring all people at risk of poverty up to the threshold, where a household is at risk if they are below the line in at least 5% of simulations, and the shortfall is the average amount that they are below the line. We report subsistence poverty, which is defined as the number of person–months spent below the food threshold, which is the minimum amount needed to meet basic food needs (18,126 PHP for the Philippines) among all people. Lastly, we report the squared poverty gap, which places a higher weight on the poverty of the poorest households and thus captures the extent, or depth, of poverty among households below the line. We use the Foster–Greer–Thorbecke (FGT) measure with α = 2 as a measure of severity of poverty. This is defined as (43)


$${\text{FGT}}_{2}=\frac{1}{6n}\overset{q}{\underset{i}{\sum }}{\left(\frac{y^{*}-y_{i}}{y^{*}}\right)}^{2}$$

for *q* person–months under the line of poverty, with the income *y*_*i*_ for person–month *i*, the total population *n*, and threshold y^*^ (2,080 PHP per month).

We convert poverty into monetary terms in order to include it in the SWF. Falling below the poverty line can be a consequence of lost income during a health crisis, but a simple estimate of income lost over the months spent in poverty would not capture the costly long-term consequences associated with persistent poverty. Persistent poverty describes a state of low income from which it is difficult to escape due to a combination of economic, social and structural factors. We place a monetary valuation on poverty to represent the possibility that short-term poverty leads to low-income households selling assets, forfeiting a future revenue source on which they would have relied to exit poverty in the longer term (44). While there is much documentation of the personal and societal costs of populations living below the line of poverty (45, 46), the costs are not readily monetised for inclusion in an objective function such as ours. We therefore consider a range of valuations: from 0 (to represent absence of poverty from the welfare function), to a high valuation of 82,000 PHP per person–month spent below the line of poverty, equal to the value of education we are using. We take this to be an upper bound because, in some low-income households, the need to support the family leads to some children leaving school (47). In these cases, the loss of one child's education is offset against the livelihood of the household as a whole, such that the benefit is shared (numerically speaking, divided) among them. For each simulated epidemic, we estimate the number of people who fall below the line of poverty in each month using a microsimulation model, and multiply that estimate by the chosen poverty valuation.

## 4 Results and discussion

We report outcomes optimized under the six objective functions in Table 2. The objective functions reflect alternative preferences for the components of welfare as defined in Equation 1. Numbers are given in units of trillion PHP. For reference, the GDP for the same period (April to September) in 2019 was 9.66 trillion PHP (see Supplementary material C.3). The resulting configurations and accompanying epidemic trajectories are presented in Supplementary Figures A1 and A2.

**Table 2:** Results from optimal configurations for six objective functions, summed over six months.

**Objective function**	**Education loss (months)**	**GDP loss (%)**	**Years of life lost (thousands)**	**Poverty (million person–months, excess)**
1 : Max GDP	2.52	11.5	239	28.4
2 : Min poverty	2.52	14	237	20.6
3 : Max SW, zero poverty weight	0	14.5	219	46.1
4 : Max SW, low poverty weight	0	14.7	221	37.7
5 : Max SW, high poverty weight	0.14	14.6	228	28.8
6 : Max SW, zero GDP weight	0	17.8	216	33.6

Education loss: Total effective months of education lost, where one month of in-person teaching contributes one month of education, and one month of remote teaching contributes 37% of one month of education.

GDP loss: Gross value added summed over all months and all sectors subtracted from pre-pandemic GDP, reported as a percentage of the pre-pandemic GDP.

Years of life lost: Total years of life lost as a result of deaths, as estimated in the epidemic model, taking into account expected years of life remaining by age group.

Poverty (excess): Millions of person–months spent below the line of poverty as a consequence of closures; this only counts person–months “in excess” of pre-pandemic person–months. Objectives 3, 4 and 5 assume zero, 10,000 PHP, and 82,000 PHP long-term loss for each person–month spent in poverty, respectively, with objective 6 using the same weight as objective 4.

The singular objective of maximizing GDP (objective 1) results in a configuration that gives a GDP of 88.5% of pre-pandemic levels, a shortfall of 11.5%. It also yields 2.52 months of education lost over the 6-month period (equivalent to full suspension of on-site schooling), 239,000 YLL, and 28.4 million person–months of excess poverty. The epidemiological constraints to the GDP maximization result in some GDP loss, and prevent an even higher YLL toll. A singular objective of minimizing poverty (objective 2) gives similar results, because it remains optimal to leave hospital occupancy at its capacity and keep schools closed. In contrast, there are only 20.6 million person–months of excess poverty and some sacrifice of GDP, with economic output at 86%. When minimizing societal welfare loss (objectives 3 to 6), education is valued as part of the objective function. The optimal configurations for all objectives except objective 5 leave schools completely open during term time (four months out of the six-month projection horizon). When poverty is valued highly in the SWF (objective 5), education loss is equivalent to 0.14 months or less than a week. Objectives that maximize societal welfare favor configurations that spare schools from closures because of the high valuation of education in the SWF. Similarly, when YLL are valued as part of the objective function, as in objectives 3 to 6, the number of YLL is lower than for the pure GDP maximization and poverty minimization objectives, ranging between 216 and 228 thousand YLL compared to 239 and 237 thousand. The YLL depend on the valuations of other dimensions of welfare in the SWF across objectives 3 to 6, notably the valuation of poverty. If poverty is excluded from the SWF (objective 3), excess poverty is highest at 46.1 million, compared to a low of 20.6 million when poverty minimization is the sole objective.

### 4.1 Other outcomes

Table 3 shows other related outcomes from the models that are not included explicitly in the objective function. As expected, numbers of COVID-19 deaths mirror numbers of YLL, and the other four measures of poverty mirror excess poverty in Table 2. The depth of poverty under each different configuration is shown in Supplementary Figure A.3.

**Table 3:** Other outcomes from optimal configurations summed over six months.

**Objective function**	**Deaths (thousands)**	**Poverty (total)**	**Poverty shortfall (billion PHP)**	**Subsistence poverty (total)**	**Squared poverty gap (%)**
1 : Max GDP	14.3	150	142	100	4.54
2 : Min poverty	14.2	142	122	84.4	3.74
3 : Max SW, zero poverty weight	12.8	168	180	129	6.01
4 : Max SW, low poverty weight	13	160	167	118	5.39
5 : Max SW, high poverty weight	13.4	151	145	100	4.52
6 : Max SW, zero GDP weight	12.7	155	159	111	5.04

Poverty (total) shows million person–months spent below the line of poverty in total, including those who would be below the line of poverty even without sector closure. The poverty shortfall—the amount by which the population is short of the poverty threshold in all months in simulations—ranges from 122 to 180 billion PHP across objectives, corresponding to 142–168 million person–months.

The percentage of person–months spent in poverty below the food threshold of 18,126 PHP shows households below the minimum income level needed to meet basic food needs. Values range from 60% to 76%, increasing as the valuation assigned to poverty in the objective function decreases. That is, policies that reduce excess poverty have the effect of reducing subsistence poverty at the same time.

Recall that the Social Amelioration Program was introduced in the Philippines at the start of the pandemic, with planned payments of 10,000 to 16,000 PHP to be made to 18 million households (48). By November 2020, 182.9 billion PHP (out of a budget of 206.7 billion PHP) had been disbursed (25). Note that this amount is, incidently, similar to the maximum poverty shortfall we project under pandemic mitigation that ignores poverty.

### 4.2 Opportunity costs of averting poverty

Comparing optimal configurations for objective 2 (minimizing poverty) with objective 1 (maximizing GDP), 7.8 million person–months of poverty are averted at a cost of 2.5% of GDP, or 245 billion PHP. This equates to a cost of about 31,200 PHP per person–month of poverty averted with the poverty minimizing objective. Additionally, 15.6 million person–months spent below the line of subsistence poverty are averted, corresponding to about 15,700 PHP per person–month of subsistence poverty averted. The opportunity costs of averting person–months below either threshold equates to about 10,000 PHP. These costs are high in the context of the Philippine GDP per capita at around 180,000 PHP. They are also high compared with the monetary value by which households fall short of the threshold for poverty, which has order of magnitude 1,000 PHP per person–month. Overall, the poverty-minimizing configuration has a poverty shortfall that is smaller by 20 billion PHP than the GDP-maximizing one (Table 3), implying that it costs 245 billion PHP to increase the income of the poorest households by 20 billion PHP with the second optimization objective. This implies that the opportunity costs of following the poverty minimizing objective are high, specifically in terms of GDP loss incurred.

Comparing objectives 3 and 6 (effectively exchanging GDP for poverty in the SWF) yields a similar value of 10,000 PHP per person–month of excess poverty averted, with an incremental GDP loss of 3.3%, and an additional 3,000 discounted YLL averted, 12.5 million person–months below the poverty line averted, and 18 million person–months below the subsistence poverty line averted.

Comparing objectives 3 and 4 (no poverty vs low poverty valuation in the SWF) yields a value of 1,400 PHP per person–month crossing a threshold, with an incremental GDP loss of 0.2%, 2,000 additional YLL, 8.4 million additional person–months below the poverty line averted and 11 million person–months below the subsistence poverty line averted. We note that the cost associated with proceeding to the next level (low poverty vs high poverty valuation, objectives 4 vs 5) is much steeper, at 10,000 PHP per person–month.

Using the SWF to find optimal configurations, we see poverty trading off primarily with YLL (objectives 3, 4 and 5). Our results imply that a reduction in excess poverty of 17.3 million person–months is exchanged for 9,000 YLL when poverty is assumed to have a high long-term costs of 82,000 PHP. At the highest valuation, there is also a trade-off with education, but not with GDP. Irrespective of the valuation of poverty, GDP loss is similar to the poverty-minimizing configuration (objective 2), with an incremental loss of about 3% compared to the GDP-maximizing configuration (objective 1).

### 4.3 Comparison with the first six months of COVID-19 in the Philippines

Over the projection horizon, schools were entirely closed, which meant that the effective education delivered was equivalent to 1.48 months. From GVA data, we estimate that GDP was 85.9% of its maximum possible value. From fitting the model to hospital data, we estimate that there were 11.4 thousand deaths and 61.2 million excess person–months spent in poverty. These outcomes are not directly comparable to our model results because we assumed transmission to be the same as that observed in March 2020. This would be a reasonable assumption to make when planning outbreak response. When fitting the model to data for the projection horizon, we find the transmission was lower by 20% on average over the period compared to March.

Even though the model inputs were different, the outcomes are similar. From this, we might have concluded that the government response was not optimal: closure options were available that would have been less costly to society in terms of economic and education losses than the ones chosen, while still resulting in fewer deaths. However, there is reason to believe that the model outcomes could have been a result of inadequate contextualization of certain model parameters. While our results are internally comparable (in that we can meaningfully compare them to each other), we find that they do not correspond well to the reality. We discuss the reasons for and consequences of this in Section 5.

## 5 Conclusions

There is an inherent trade-off between societal outcomes when mitigating pandemics with non-pharmaceutical interventions between economic activity and education on the one hand and averting deaths and sickness on the other. There is also a trade-off between different economic sectors, in that closing some might allow others to remain open. Here, we use an economy-structured epidemiological model to explore the trade-off between GDP, health and educational losses, and the number of people who experience poverty due to mandated business closures.

There are numerous challenges in undertaking this type of modeling work. The biggest obstacle to modeling of this kind is the paucity of relevant parametrising data, in particular pertaining to workplace contact rates, which leads us to borrow values from a study carried out in France (49) and previously applied to the UK (29). We are not aware of data on sector-specific contact rates pertaining to any setting other than France and the UK (50), and this greatly inhibits the potential to jointly model epidemics and economics (51). As discussed in Section 4.3, our model is inadequately contextualized to the Philippines, limiting the prospect of developing a planning tool to assist in the management of future outbreaks (52). To this end, country-specific data are essential. Absent an appropriately parametrised model, we are limited to abstracted comparisons that shed light on dynamic trade-offs in a general sense. What we learn from this study is that, if we want to use modeling tools to provide guidance to decision makers that is relevant and actionable, then we need to invest the time and resources into acquiring the data needed to properly parametrise the model in context. Data collection should hence be consistent and timely in order to be prepared for the next outbreak.

Our economic projections are static and not dynamic: there is no relationship between the probability to lose income from one month to the next, and there are no changes to sector of employment or sector sizes over time. This might be a particularly poor assumption for informal workers, who are more likely to lose their primary source of income and eventually find work in another sector, potentially avoiding income loss and keeping them above the line of poverty. There are dynamic feedback interactions among the processes we aim to recapitulate that we cannot capture without a full macroeconomic model. For example, we have not taken account of macroeconomic impacts such as changes in income, savings, international trade and investments. Instead, we focus on what we believe are the most important aspects to capture and are most relevant in the short time horizon of six months.

We note the challenges inherent in working with a VSL to value life. It serves a purpose here in making explicit the trade-off between competing aspects of societal welfare. A particular difficulty it highlights, one which has not been resolved and to which we would like to bring attention, is that of distribution. We use a single, population-average VSL. In principle, the VSL reflects willingness to pay and, in practice, it is commonly a function of average income (32). This implies that households with a lower income, who have a reduced willingness to pay as a consequence of having a reduced ability to pay, might be required, through mandated closures, to sacrifice their income to a cost that exceeds what they would choose to pay to avert those risks to their own mortality. Situations such as these have been described by Sunstein as detrimental to their welfare (53). The policy therefore delivers welfare to some in the population at the expense of others. This inequality is compounded twice: once, as the cost (in terms of lost income) falls disproportionately on those with lower incomes [who are more likely to lose employment (54)], and again, with those with lower incomes likely having worse health outcomes in the pandemic (55). The VSL illustrates this compounded injustice, and potentially offers a means to redress it. However, as (53, page 90) writes, “Whether government should use a higher or lower VSL across demographic lines cannot be answered simply. Any judgment about the appropriate VSL, and about individuation, must be heavily pragmatic. It must rest on the human consequences for one or another choice.” If a policy consistent with a lower VSL would not be acceptable, then redistribution of income from the wealthiest households to those at risk of poverty is justified.

We have particular difficulty valuing poverty, which we would do by quantifying the longer-term cost of persons falling into poverty. We could use estimates of the long-run costs of childhood poverty presented in Clarke et al. (56), but we would need to account also for costs of adults in poverty, and the possibility of persistent poverty occurring as a consequence of the epidemic-induced hardships. With no published estimates, we considered two values, one chosen to be equivalent to the cost of missed education , which we take to be an upper bound, and one chosen to be smaller. Education and poverty are closely related, and the choice of which workers retain their jobs may also impact long-term education outcomes, as maintaining incomes among low-income households may have the benefit of keeping students in school: historically, the main reason for students in the Philippines to drop out of school was to work (47).

We use an optimisation approach in order to compare extreme outcomes: the smallest possible GDP loss vs. the smallest possible poverty impact. A major limitation of optimisation is that it is incompatible with uncertainty: we assumed a single level of transmission for the period, and we evaluated expected outcomes of the microsimulation model. This means we do not gain a sense of the distribution over likely outcomes. Instead, we learn the differences between “best case” scenarios, under the assumption that transmission takes a particular value.

An alternative to attenuating transmission at the expense of subjecting households to months of poverty exists in providing targeted financial support through welfare transfers. We considered how redistribution could be used to achieve comparable outcomes in terms of averting poverty and protecting GDP. Using the LFS and FIES data, we estimated that, at the upper end of the income spectrum, there are around 10 million households whose income is never below the line of poverty no matter the closure of the sectors in our scenarios (Supplementary Figure S2). We estimate that the amount by which their per-person non-work and agriculture- and education-sector incomes exceed the threshold for poverty is around 720 billion PHP over the six-month period. Administration costs notwithstanding, redistributing 25% of this income (180 billion PHP) of those not at risk of poverty would keep all those in poverty or at risk due to closures above the line of poverty in the case that the three-component social welfare function is maximized. Thus, effective redistribution could be used to address poverty which would allow closures to be used to mitigate the epidemic. Modeling such a policy would require consideration of its impacts on GDP in terms of consumption in the near term and on inflation and debt obligations in the longer term.

We estimate the opportunity cost of averting poverty to be high. If, like education, failure to protect households from falling into poverty results in costs that accumulate over the duration of a lifetime, then the opportunity cost of *not* protecting households from months of poverty will also be high. Making the case for the distribution and redistribution of income in epidemic mitigation will turn on the same arguments of moral imperatives and political, fiscal, administrative and other constraints as in “normal” times, where households suffer unexpected financial hardship. The difference in an epidemic scenario is the macroeconomic impact that comes from so many households experiencing financial uncertainty and income losses at the same time. Viewed from this angle, we conclude that ongoing efforts to reduce poverty constitute pandemic preparedness in the sense that they build society's resilience to disasters (57, 58).

## Data Availability

The original contributions presented in the study are included in the article/Supplementary material.
