# Supplementary material for: Competing societal objectives in epidemic mitigation: a modeling study of COVID-19 in the Philippines
Source: Front Public Health. 2025 Nov 3;13:1662043. doi: 10.3389/fpubh.2025.1662043 (PMC12620433; doi:10.3389/fpubh.2025.1662043)
Supplement: Supplementary file 1 [file Data_Sheet_1.pdf]

Competing societal objectives in epidemic mitigation: A modelling study of COVID-19 in the Philippines – Supplementary Online Material

Table of Contents

A Supplementary figures **2**

A.1 Configurations 7

A.2 Epidemic trajectories 9

A.3 Distribution of shortfall for the month of April 11

B Population data **13**

B.1 Population age distribution 13

B.2 Life expectancy 14

C Economic data **15**

C.1 Survey data 15

C.2 Sector summaries 19

C.3 Sector outputs by month 23

D Epidemic data **26**

D.1 Cases, hospitalisations and deaths 26

D.2 Hospitalisation and fatality rates 28

D.3 Other parameters 29

E Epidemic model **30**

E.1 Contact rates 30

E.2 State transitions 35

E.3 Transition rates 36

F Fitted parameters **37**

A Supplementary figures

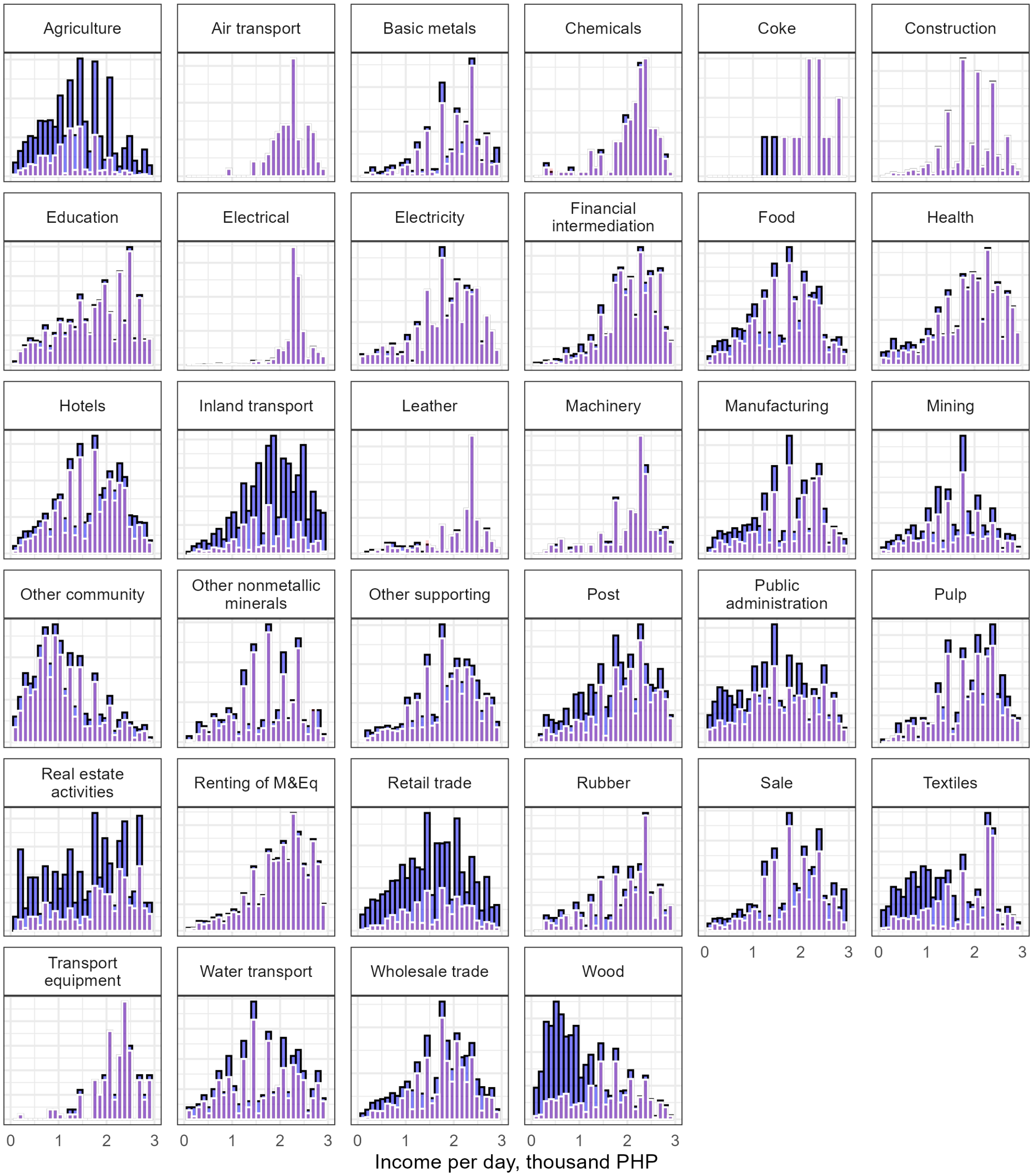

Figure S1: Distribution of basic daily income by sector, Labour Force Survey (LFS), Philippine Statistics Authority (2018b). White/foreground: raw values. Black/background: raw and imputed values.

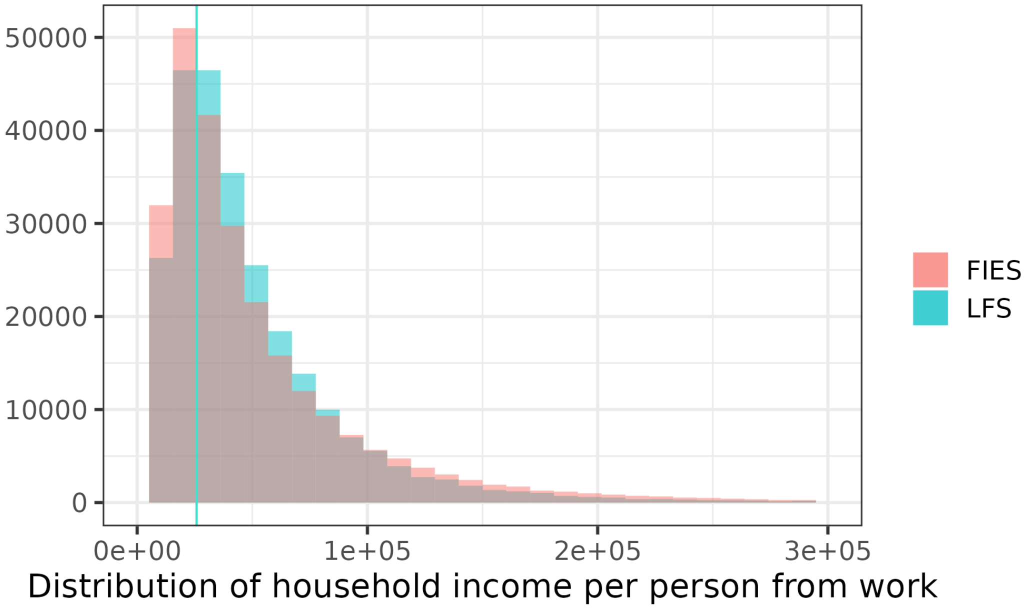

Figure S2: Distribution of per-person household income from work-related income, showing raw values from the Family Income and Expenditure survey (FIES), Philippine Statistics Authority (2018a) and values imputed using LFS data. The threshold for poverty is shown in turquoise.

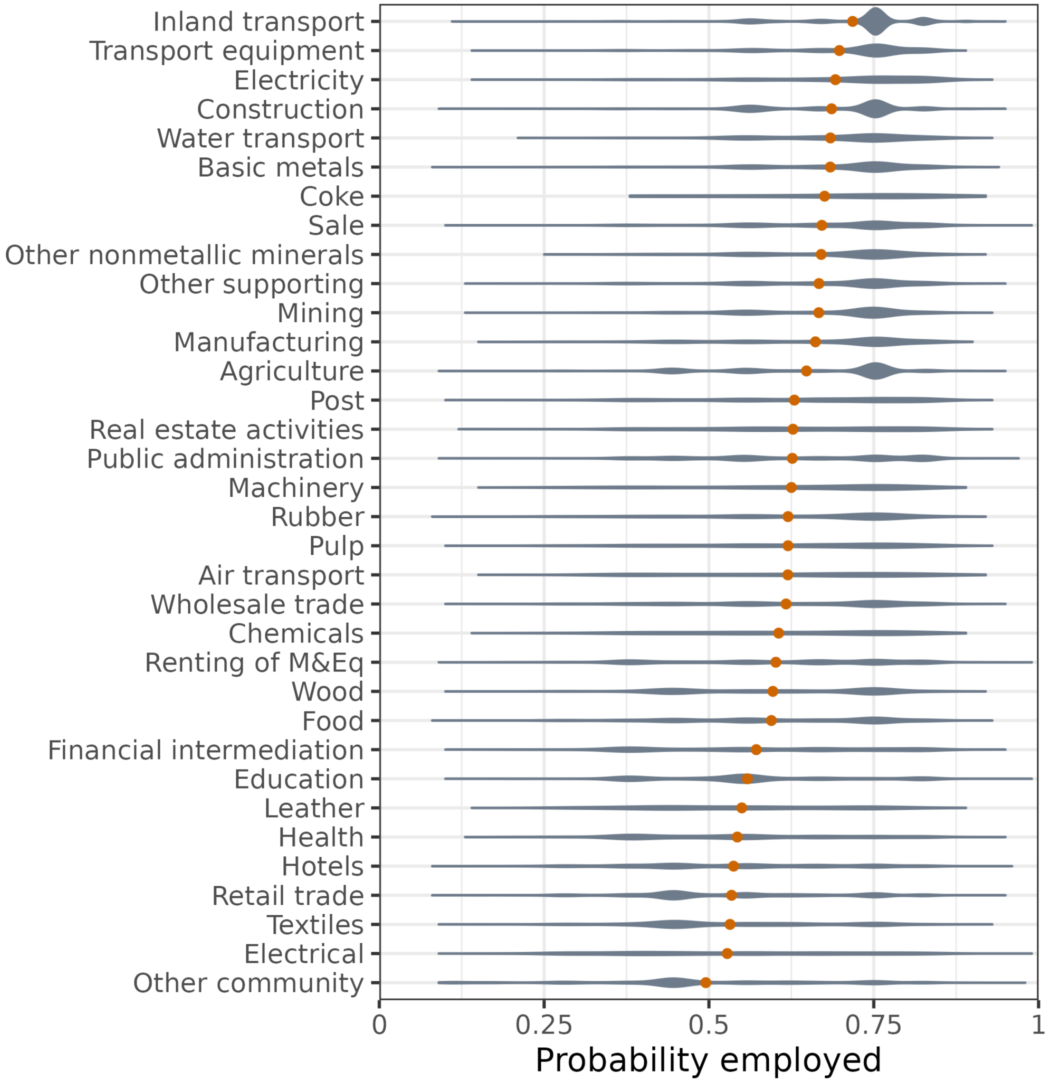

Figure S3: Unscaled probabilities for individuals to keep their jobs by sector. Distributions are shown in grey. Sector averages are shown in orange.

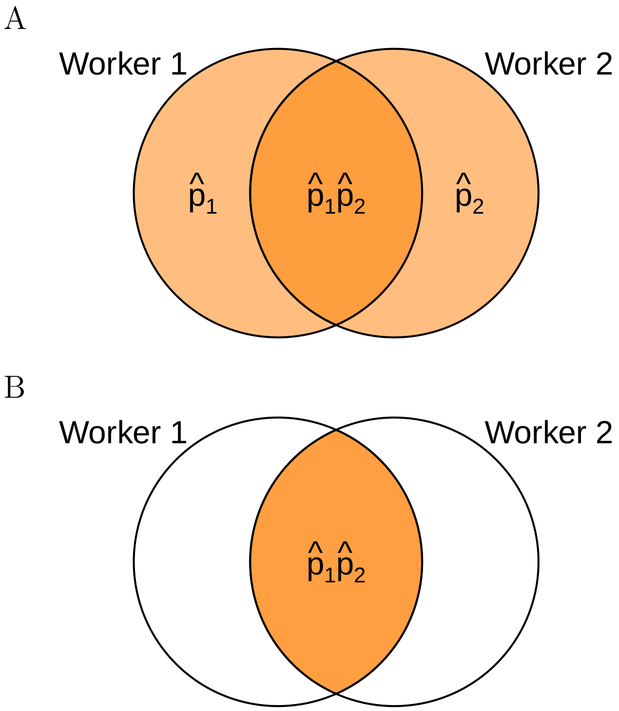

Figure S4: Visualisations of $f_h()$ in the case that there are two workers in the household whose incomes might be necessary for the household to stay above the line of poverty. $f_h()$, the probably that the household retains sufficient income to remain above the line of poverty, appears in the equation to estimate the total amount of poverty. Here, there are two possible expressions for $f_h()$: A: Either worker's income will be sufficient to keep per capita household income above the line of poverty: $f_h = \hat{p}_{\tau,h1} + \hat{p}_{\tau,h2} - \hat{p}_{\tau,h1}\hat{p}_{\tau,h2}$. B: Both workers' incomes are required to keep per capita household income above the line of poverty: $f_h = \hat{p}_{\tau,h1}\hat{p}_{\tau,h2}$.

A.1 Configurations

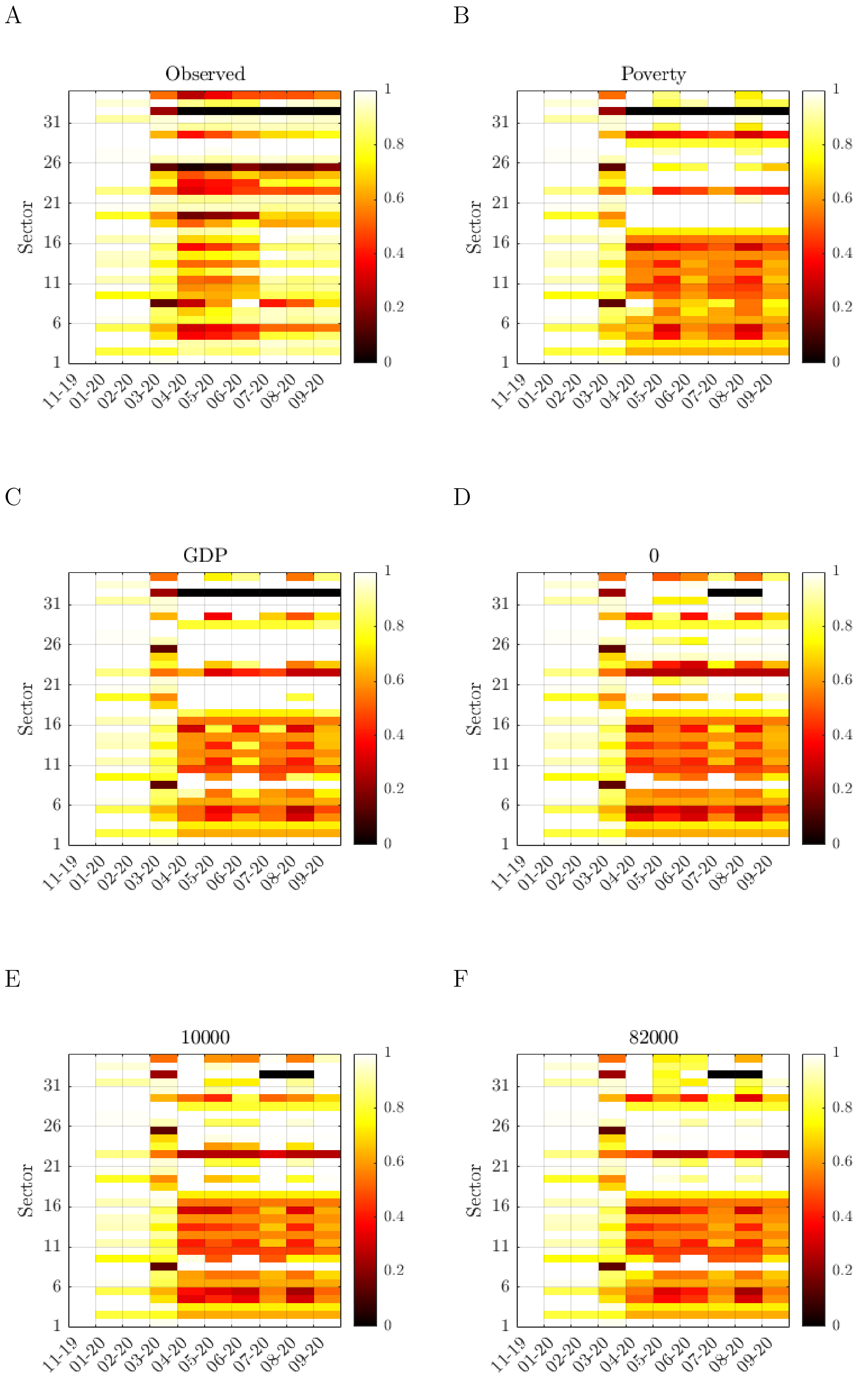

Figure S5: Economic configurations. A: the configuration we estimate using observed GVA per quarter.

[illegible]

A.2 Epidemic trajectories

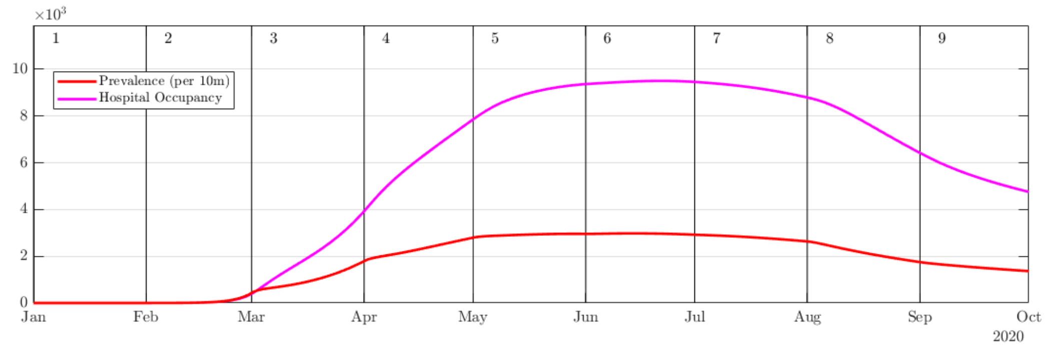

Figure S6: Epidemic trajectories for the configuration that minimises poverty.

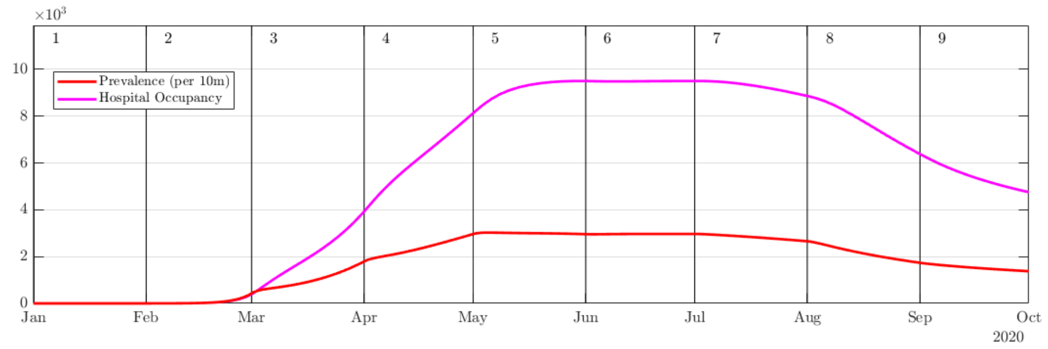

Figure S7: Epidemic trajectories for the configuration that maximises GDP.

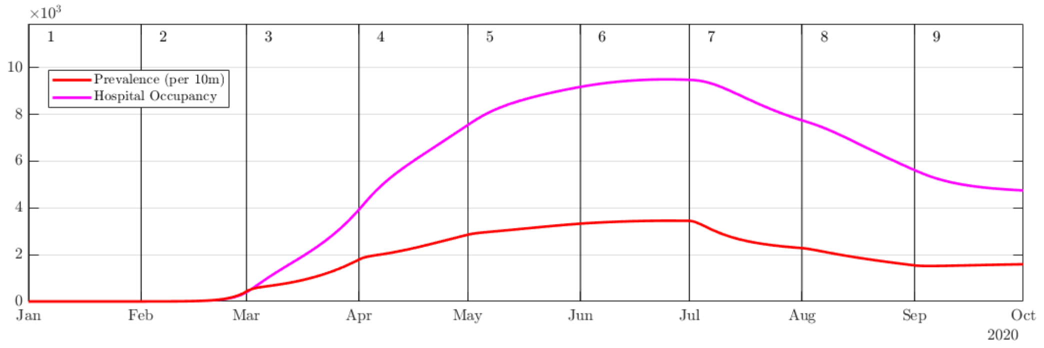

Figure S8: Epidemic trajectories for the configuration that maximises the three-component social welfare function.

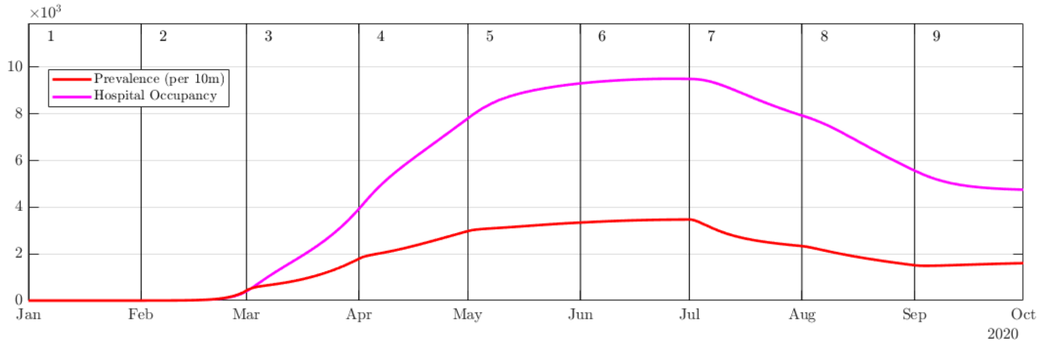

Figure S9: Epidemic trajectories for the configuration that maximises the four-component social welfare function with low poverty weight.

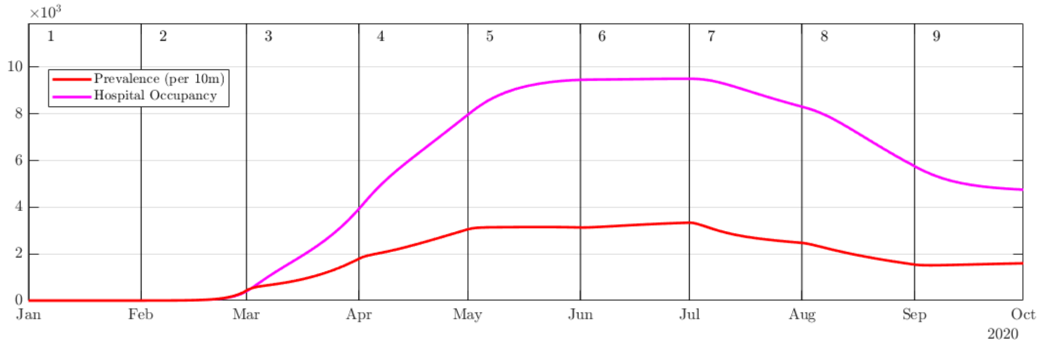

Figure S10: Epidemic trajectories for the configuration that maximises the four-component social welfare function with high poverty weight.

A.3 Distribution of shortfall for the month of April

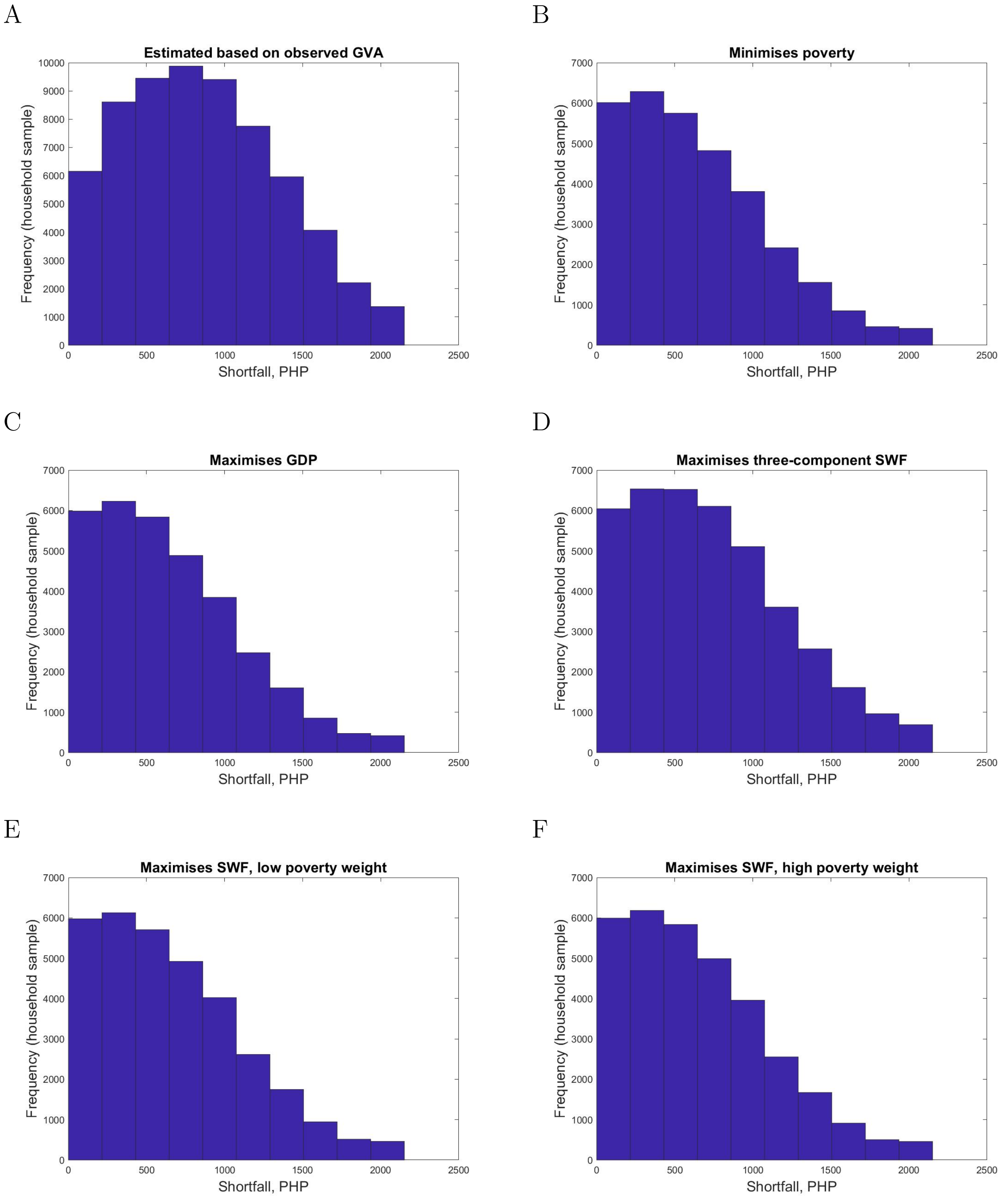

Figure S11: Distribution of per capita household income as a shortfall from the line of poverty. A: the shortfall we estimate using observed GVA per quarter. B to F: distributions from configurations that optimise the SWF for each objective. B: minimising poverty. C: maximising GDP. D: three-component SWF. E: four-component SWF with low poverty weight. F: four-component SWF with high poverty weight.

B Population data

B.1 Population age distribution

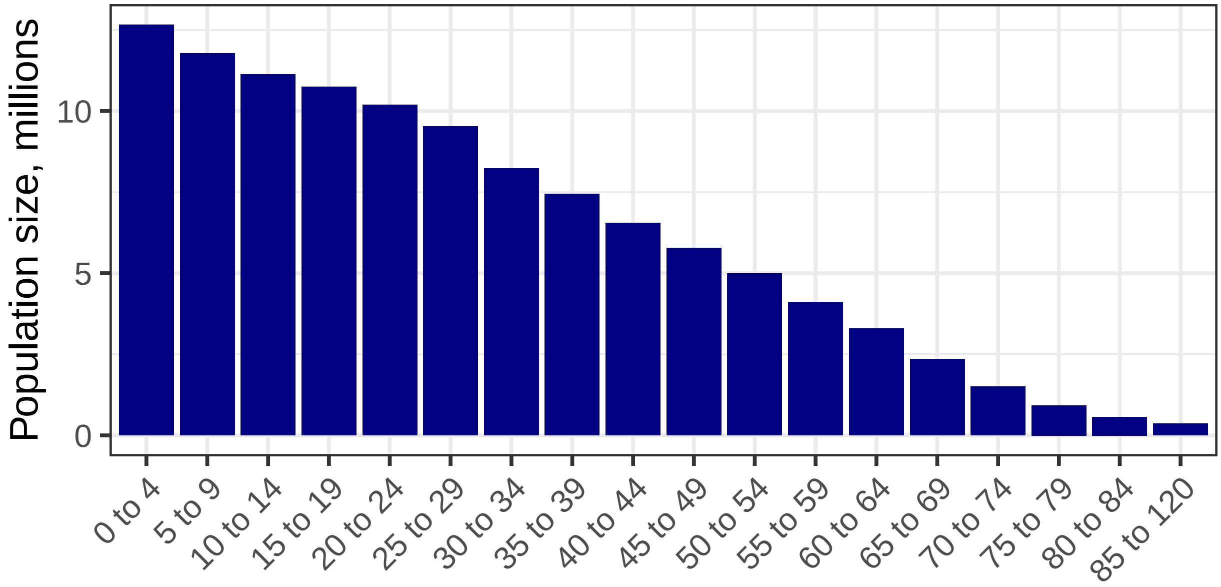

Figure S12: Number of people in each five-year age band in the Philippines for the year 2019 (Global Burden of Disease Collaborative Network, 2021)

The DAEDALUS model is a stratified compartmental model, where each person belongs in one group. It uses four age groups, with the working-age age group further subdivided into those working and those not working. Population counts from IHME (Figure S12) are collapsed into DAEDALUS's four age groups as shown in Table S1. The working-age population is split into those working and those not. Here, 42% of the working-age population are modelled as not working.

Table S1: Number of people in each age group, and number of people of working age who work, for the DAEDALUS model of the Philippines.

Group	Population
0 to 4	12,654,309
5 to 14	22,912,102
15 to 69, economically inactive	30,781,314
70 to 120	3,366,675
15 to 69	73,209,679

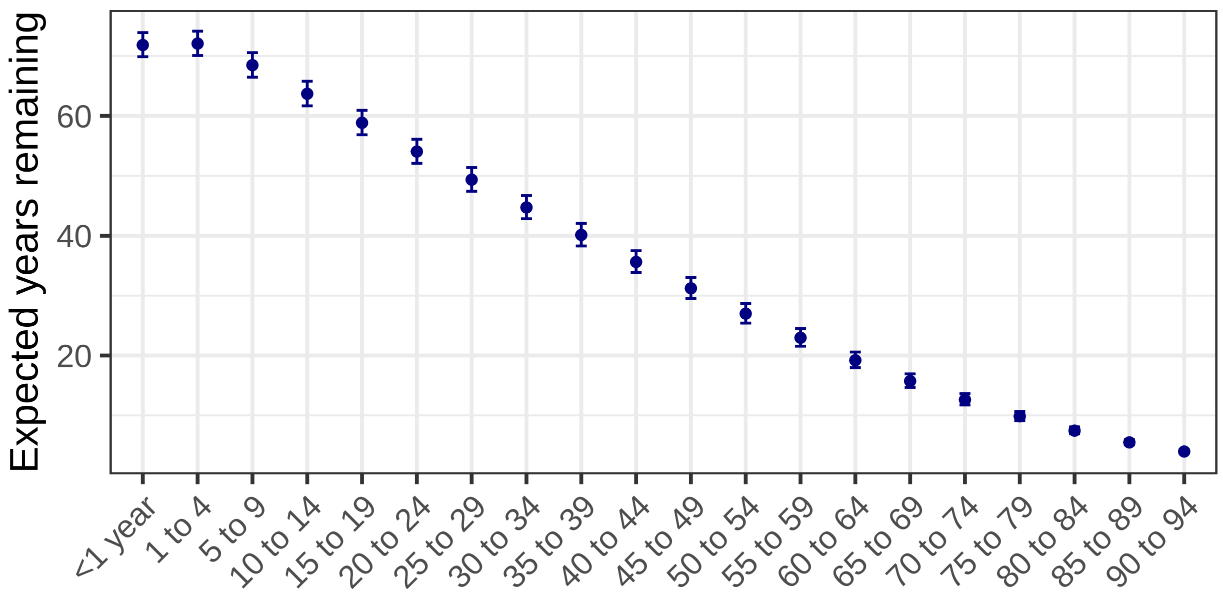

Figure S13: Expected years of life remaining (l_a) by age for people living in the Philippines for the year 2019 (Global Burden of Disease Collaborative Network, 2021)

B.2 Life expectancy

We use Global Burden of Disease (GBD) estimates of remaining life expectancy (Global Burden of Disease Collaborative Network, 2021). We map the remaining life expectancy l_a from the GBD age groups, which are in five-year bands and denoted by a, to the DAEDALUS model age groups (0 to 4, 5 to 14, 15 to 69, and 70 and older), denoted by g. We take the population-weighted average for each group g, taking into account the population size of each age group, N_a.

B.2.1 Value of a discounted life year

For life expectancy by age groups g, we have

$$l_g^{(\text{life})} = \frac{\sum_{a \in g} N_a l_a}{\sum_{a \in g} N_a}. \tag{6}$$

To translate remaining life expectancy to the number of discounted life years, we make an approximation using the geometric sum of discounted values added up over years. Because the remaining life expectancy need not be an integer number of years, and the act of discounting applies discretely to years, the final fraction of a year should be added with full discounting:

$$\hat{l}_g^{(\text{life})} = \sum_{y=1}^{\lfloor l_g^{(\text{life})} \rfloor} \frac{1}{(1+r)^y} + \left(l_g^{(\text{life})} - \lfloor l_g^{(\text{life})} \rfloor \right) \frac{1}{(1+r)^{\lceil l_g^{(\text{life})} \rceil}}. \tag{7}$$

Using the identity that

$$\sum_{y=1}^{n} \frac{1}{(1+r)^y} = \frac{1-(1+r)^{-n}}{r},$$

for integer n, we make the approximation

$$\hat{l}_g^{(\text{life})} \approx \frac{1-(1+r)^{-l_g^{(\text{life})}}}{r} \tag{8}$$

The deviation of Equation 8 from Equation 7 is at most 0.014% (8.627 vs. 8.628 discounted life years remaining for people aged 70 and over), and at most 7.8×10^{-5}% (for people aged 0 to 4).

Finally, the value of a discounted life year (VdLY) can be estimated using the VSL and the remaining life expectancy for the population by age group:

$$\text{VSL} = \frac{\sum_g N_g \hat{l}_g^{(\text{life})}}{\sum_g N_g}\text{VdLY}.$$

B.2.2 Number of discounted life years lost

To estimate the expected number of life years lost per COVID-19 death, $l_g^{(\text{death})}$, we follow the same method as for Equation 6 and take into account the probability to die given infection, $P(D|I,a)$:

$$l_g^{(\text{death})} = \frac{\sum_{a\in g} N_a l_a P(D|I,a)}{\sum_{a\in g} N_a P(D|I,a)}.$$

Then the expected life years lost due to a COVID-19 death, with discounting taken into account, can be approximated as

$$\hat{l}_g^{(\text{death})} \approx \frac{1-(1+r)^{-l_g^{(\text{death})}}}{r} \tag{9}$$

for discount rate $r > 0$. The total number of discounted life years lost given D_g deaths due to COVID-19 (a direct output from the epidemic model) for each age group is

$$L = \sum_g D_g \hat{l}_g^{(\text{death})}.$$

C Economic data

C.1 Survey data

We use two surveys from 2018 to construct the synthetic population that forms the model for estimating poverty as a function of sector closures.[1] The surveys are the Family Income and Expenditure Survey (FIES, Philippine Statistics Authority (2018a)) and the Labour Force Survey

[1]The surveys were made available by the Philippines Statistics Authority, https://psa.gov.ph/terms-of-use

(LFS, Philippine Statistics Authority (2018b)). The surveys are linked in the sense that they represent the same households: all household members are surveyed in the LFS and one household member is surveyed in FIES. We summarise the variables that we use from these surveys in Tables S2 and S3, respectively.

Table S2: Variables we use from the Family Income and Expenditure Survey, their data types, summary statistics, and number of observations (Philippine Statistics Authority, 2018a).

Variable name	Definition	Class	Number of categories in dataset	Mean; median (90% interval)	Number of entries
FSIZE	Family Size	Numeric		4.5; 4 (1–8)	147717
WAGES	Salaries/Wages from both Agri and Non-Agri Activities	Numeric		142032.9; 85800 (0–498811.2)	147717
EAINC	Total Income from Entrepreneurial Activites	Numeric		62930.3; 17600 (0–220998.4)	147717
TOINC	Total Income	Numeric		294179.5; 202549 (73394.8–786126)	147717
HS001005_JOB	Household Head has a job	Binary	2		147717
HS001007_IND	Kind of Industry	Categorical	491		115985
H150101_BLDG_TYPE	Building Type	Categorical	7		147717
H150103A_MAIN	Main Material of the Housing Unit	Categorical	8		147717
H150104_TENURE_STA	Tenure Status of the Housing Unit	Categorical	8		147717
V940060	Total Withdrawals from saving/business equity	Numeric		5290.4; 0 (0–19792)	147717
V930410	Total interest bank deposits	Numeric		153.5; 0 (0–0)	147717

Table S3: Variables we use from the Labour Force Survey, their data types, summary statistics, and number of observations (Philippine Statistics Authority, 2018b).

Variable name	Definition	Class	Number of categories in dataset	Mean; median (90% interval)	Number of entries
W_REGN	Region	Categorical	17		675473
LC03_REL	C03-Relationship to Household Head	Categorical	11		675473
LC04_SEX	C04-Sex	Categorical	2		675473
LC05_AGE	C05-Age as of Last Birthday	Numeric		29.1; 25 (3–67)	675473
LC06_MSTAT	C06-Marital Status	Categorical	6		617592
LC07_GRADE	C07-Highest Grade Completed	Categorical	515		617592
LC11_WORK	C11-Work Indicator	Categorical	2		606564
LC12_JOB	C12-Job Indicator	Categorical	2		347739
LC14_PROCC	C14-Primary Occupation	Categorical	449		259880
LC16_PKB	C16-Kind of Business (Primary Occupation)	Categorical	511		259880
LC18_PNWHRS	C18-Normal Working Hours per Day	Numeric		7.6; 8 (3–12)	258127
LC19_PHOURS	C19-Total Number of Hours Worked during the past week	Numeric		42.6; 48 (10–72)	258127
LC23_PCLASS	C23-Class of Worker (Primary Occupation)	Categorical	7		258127
LC24_PBASIS	C24-Basis of Payment (Primary Occupation)	Categorical	8		160427
LC25_PBASIC	C25-Basic Pay per Day (Primary Occupation)	Numeric		460.6; 350 (130–1114)	144592
LC28_THOURS	C28-Total Hours Worked for all Jobs	Numeric		43.5; 48 (12–72)	258127
PWGTPRV	Final Weight Based on Projection (provincial projections)	Numeric		157.7; 107.8 (30.9–480.7)	675473

C.2 Sector summaries

Table S4 below summarises the inputs for the 34 Sectors for Philippines. "Workforce" and "WFH" (the percentage able to work from home) are specific to Philippines. Contact rates M^{WW} and M^{CW} are extrapolated from (Béraud et al., 2015) assuming the UK distribution of workforce across sectors in mapping from 64 (from Haw et al. (2022a)) to 34.

Workforce data are based on the 2010 Census of Population and Housing population projections. They come from NEDA estimates using the January, April, July, and October 2019 Labor Force Survey Public Use Files

WFH data are based on 2015 Census of Population and Housing population projections. Data do not include those who are with job but not "at work" or working. Details may not add up to totals due to rounding of figures. They come from NEDA estimates using the January, February, and March 2021 Labor Force Survey Public Use Files, who emphasise caution in utilizing the estimates due to small sample size. Some sectors have coefficient of variation greater than 20%.

Table S4: Summary of sector inputs. WFH is the percentage of workers who can work from home. M^{WW} is the contacts between workers in sectors. Contacts between workers and consumers are recorded by age in age0to4, ..., age70plus. GVA, % is the percentage contribution to total gross value added.

Sector	Workforce	WFH	M^{WW}	age0to4	age5to14	age15to69	age70plus	GVA, %
Agriculture, hunting, forestry, and fishing	9,698,304	0.2	2.73	0	0	0	0	9.2
Mining and quarrying	184,226	0.47	19.11	0.01	0	0.16	0.01	0.87
Food, beverages, and tobacco	1,111,839	1.25	19.11	0.01	0	0.16	0.01	9.6
Textiles and textile products	547,474	4.82	19.11	0.01	0	0.16	0.01	0.68
Leather, leather products, and footwear	81,387	0	19.11	0.01	0	0.16	0.01	0.08
Wood and products of wood and cork	286,451	0.35	19.11	0.01	0	0.16	0.01	0.18
Pulp, paper, paper products, printing, and publishing	151,883	0.36	14.39	0.02	0.01	0.43	0.02	0.3
Coke, refined petroleum, and nuclear fuel	7,083	21.21	19.11	0.01	0	0.16	0.01	0.9
Chemicals and chemical products	88,700	4.05	19.11	0.01	0	0.16	0.01	2.33

Sector	Workforce	WFH	M^{WW}	age0to4	age5to14	age15to69	age70plus	GVA, %
Rubber and plastics	114,944	0	19.11	0.01	0	0.16	0.01	0.21
Other nonmetallic minerals	115,315	0.63	19.11	0.01	0	0.16	0.01	0.46
Basic metals and fabricated metal	219,992	4.03	19.11	0.01	0	0.16	0.01	0.6
Machinery, nec	45,874	0	19.11	0.01	0	0.16	0.01	0.25
Electrical and optical equipment	473,648	2.66	19.11	0.01	0	0.16	0.01	2.27
Transport equipment	92,575	0.06	19.11	0.01	0	0.16	0.01	0.38
Manufacturing, nec; recycling	281,009	0.19	19.11	0.01	0	0.16	0.01	0.49
Electricity, gas, and water supply	155,584	2.04	19.11	0.01	0	0.16	0.01	3.01
Construction	4,152,742	0.42	5.46	0.01	0.01	0.32	0.02	7.78
Sale, maintenance, and repair of motor vehicles and motorcycles; retail sale of fuel	473,740	0.24	4.09	0.21	0.14	4.76	0.27	0.57
Wholesale trade and commission trade, except of motor vehicles and motorcycles	588,842	0.92	4.09	0.21	0.14	4.76	0.27	3.48

Sector	Workforce	WFH	M^{WW}	age0to4	age5to14	age15to69	age70plus	GVA, %
Retail trade, except of motor vehicles and motorcycles; repair of household goods	7,390,286	2.35	4.09	0.21	0.14	4.76	0.27	13.95
Hotels and restaurants	1,917,845	0.96	4.09	0.21	0.14	4.76	0.27	2.2
Inland transport	2,876,819	0.34	4.09	0.21	0.14	4.76	0.27	1.95
Water transport	63,101	1.06	4.09	0.21	0.14	4.76	0.27	0.21
Air transport	31,402	0	4.09	0.21	0.14	4.76	0.27	0.61
Other supporting and auxiliary transport activities; activities of travel agencies	460,496	1.01	4.09	0.21	0.14	4.76	0.27	0.91
Post and telecommunications	425,442	17.38	6.53	0.14	0.1	3.29	0.19	3.02
Financial intermediation	581,586	4.55	20.47	0.09	0.06	2.06	0.12	8.65
Real estate activities	231,656	6.24	20.47	0.09	0.06	2.06	0.12	3.56
Renting of M&Eq and other business activities	1,961,156	11.89	10.92	0.04	0.03	0.89	0.05	8.93

Sector	Workforce	WFH	M^{WW}	age0to4	age5to14	age15to69	age70plus	GVA, %
Public administration and defense; compulsory social security	2,784,847	1.24	0	0.42	0.29	9.53	0.54	4.5
Education	1,282,635	8.75	0	1.26	12.58	11.49	0.01	3.95
Health and social work	543,155	0.35	0	0.42	0.29	9.53	0.54	1.67
Other community, social, and personal services	3,006,329	0.55	8.19	0.01	0.01	0.32	0.02	2.26

C.3 Sector outputs by month

The reduction in economic output associated with the pandemic and mitigation policies is a crucial component of DAEDALUS, measured by sector gross value added (GVA) and level of closure represented by the percentage reduction in economic output, with 100% indicating the sector operating at pre-pandemic levels and 0% indicating the sector being completely closed for any production. Zero production is not observed, as even the most stringent business closures leaves essential economic activity operational. We use data on quarterly real GVA by 34 sectors to estimate the shock to the economy. Quarterly (and not monthly) data on GVA are available. Quarterly averages underestimate the shock to economic output in specific months where stringent business closures were implemented, but that were less stringent in other months of the quarter. We estimate the missing monthly values of economic output in several steps:

1. We estimate the amount the sectors were closed (the loss) in each quarter of 2020 (Figure S14)
2. We calculate a monthly economic stringency index of government restrictions on economic exchange by adding the values for the OxCGRT indicators (C2, workplace

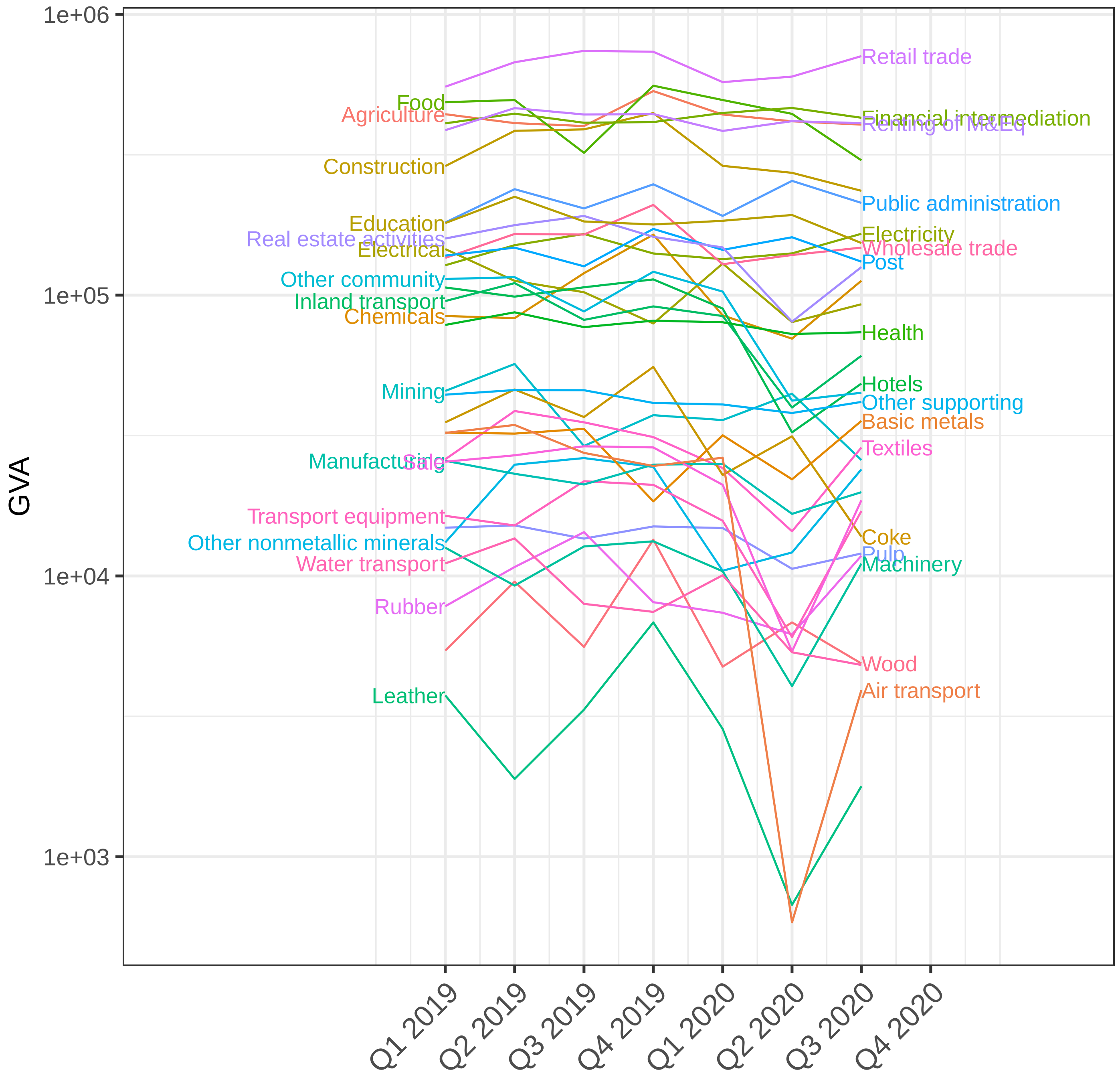

Figure S14: Reported GVA per quarter by sector at constant prices.

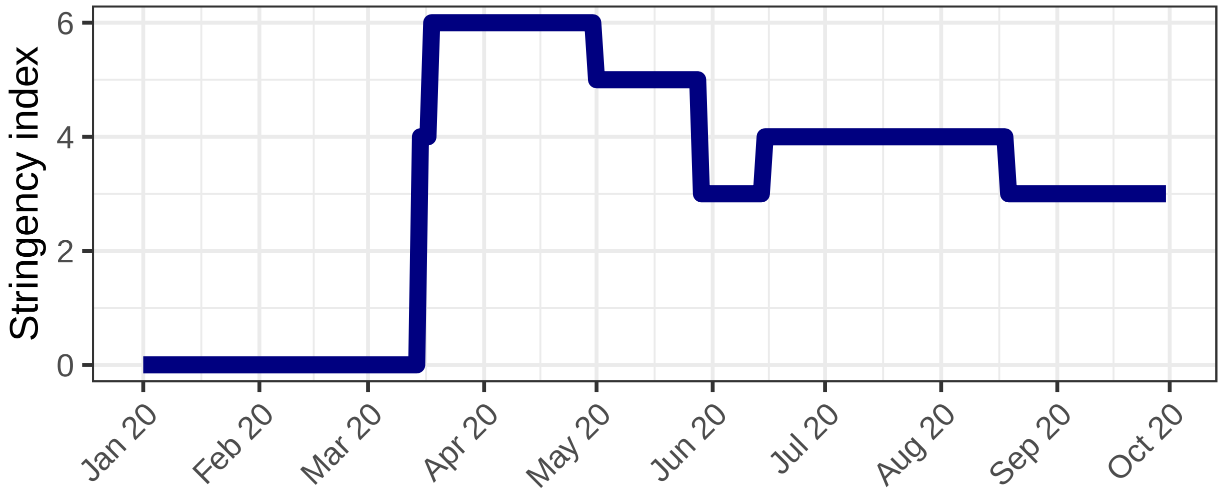

Figure S15: OxCGRT stringency index: sum of stringency indices (C2, workplace closing; C6, stay-at-home requirements) for the government's response, as measured and quantified by OxCGRT, over time.

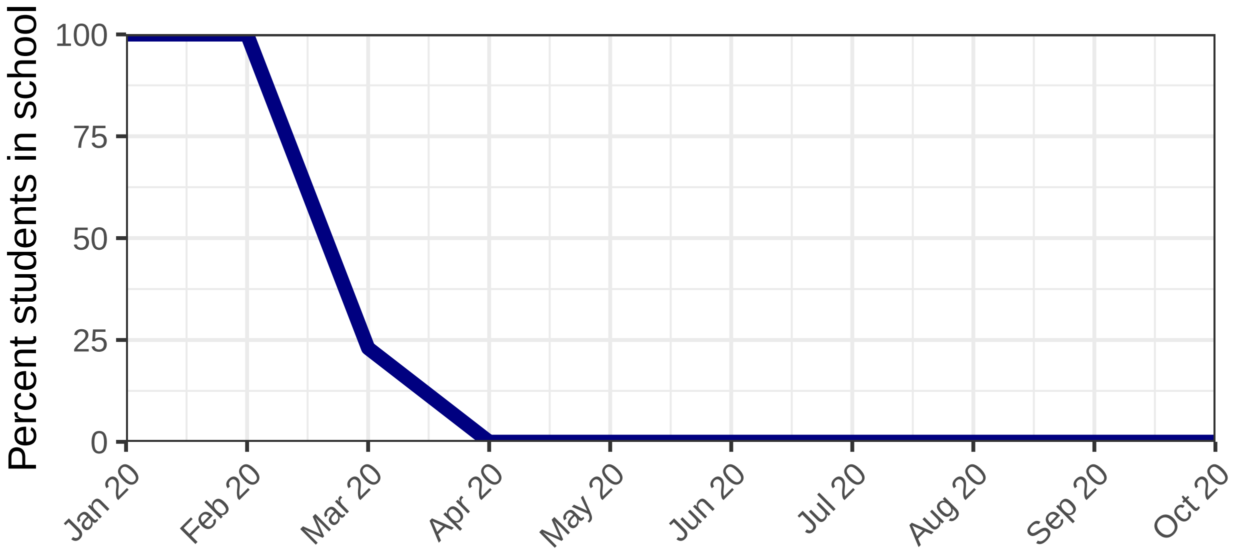

Figure S16: School opening over time from PHL NEDA team. Schools closed on 23 March 2020. Schools partially opened on 7 June 2021 - but possibly less than 1%.

closing; C6, stay-at-home requirements). This indicator is bound between zero (no restrictions) and 6 (maximum restrictions), see https://github.com/OxCGRT/covid-policy-tracker/blob/master/documentation/codebook.md (Figure S15)

3. We distribute the loss between the three months of each quarter linearly as a function of the economic stringency index.
4. We estimate monthly GVA values as one-third the quarterly GVA minus the loss for the month.
5. We estimate monthly fractions of workers working as the estimated monthly GVA divided by the maximum possible.

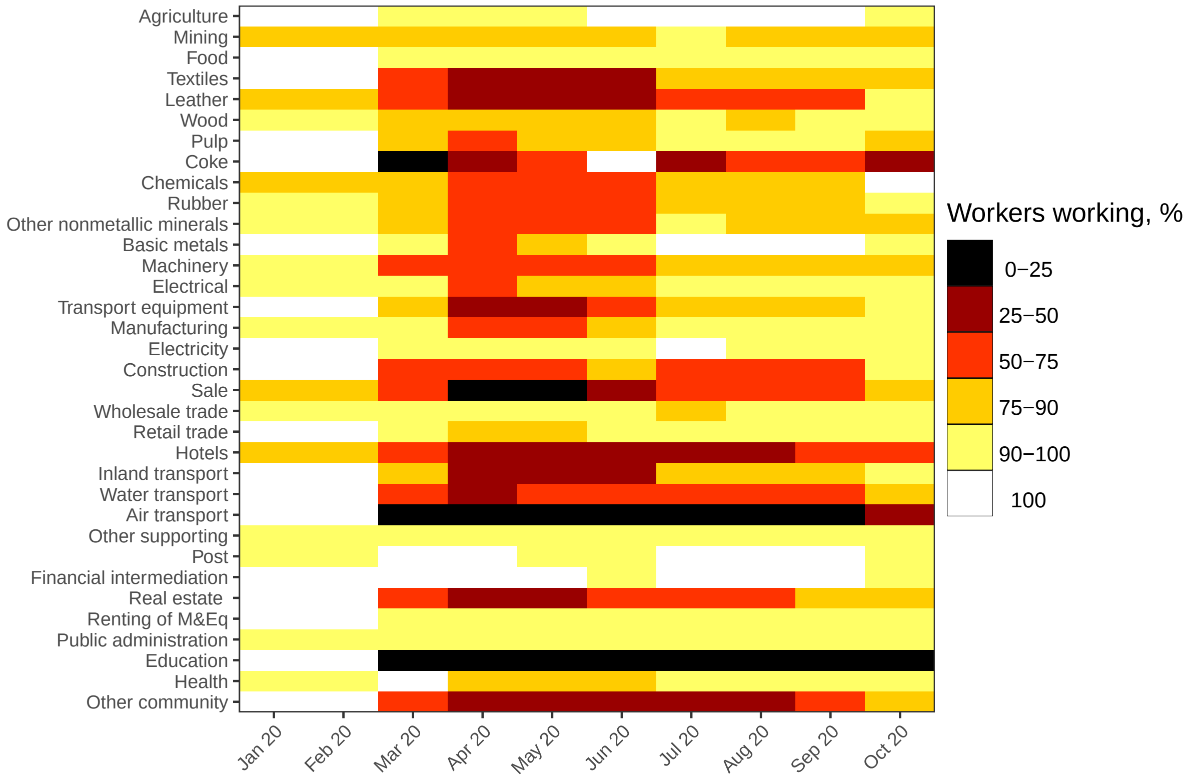

Figure S17: Estimated sector opening by month. We cap values at 100% in both the economic and epidemic parts of the model.

D Epidemic data

D.1 Cases, hospitalisations and deaths

Because hospital occupancy data start in July 2020, we use death data up to then to fit the model.

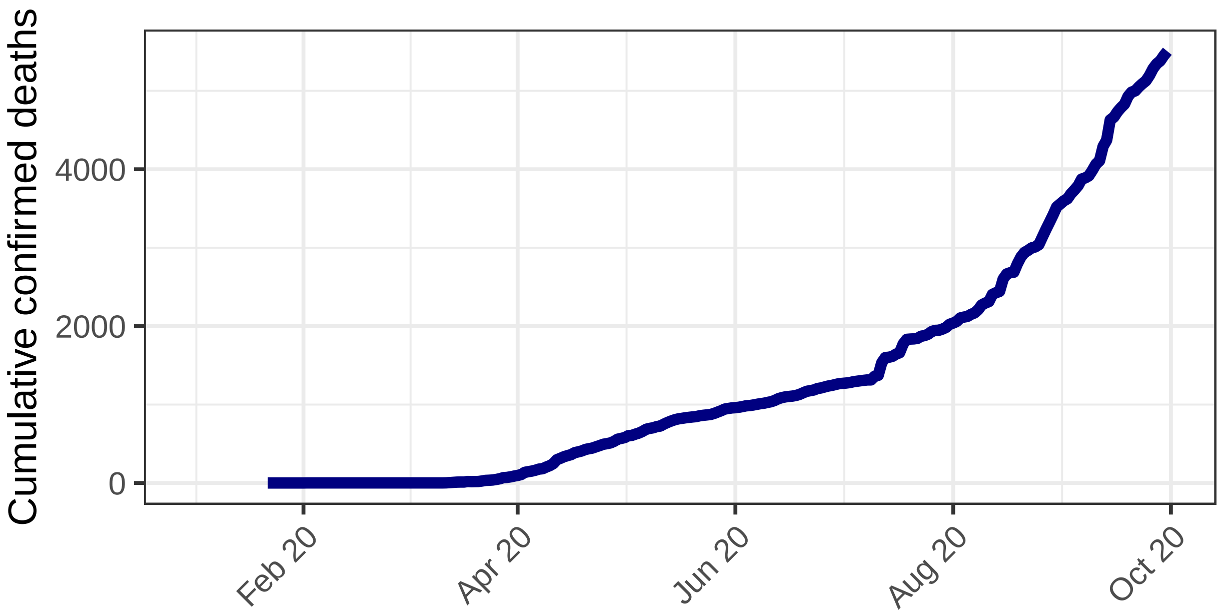

Figure S18: Official number of deaths from DOH via https://covid.ourworldindata.org (OWID)

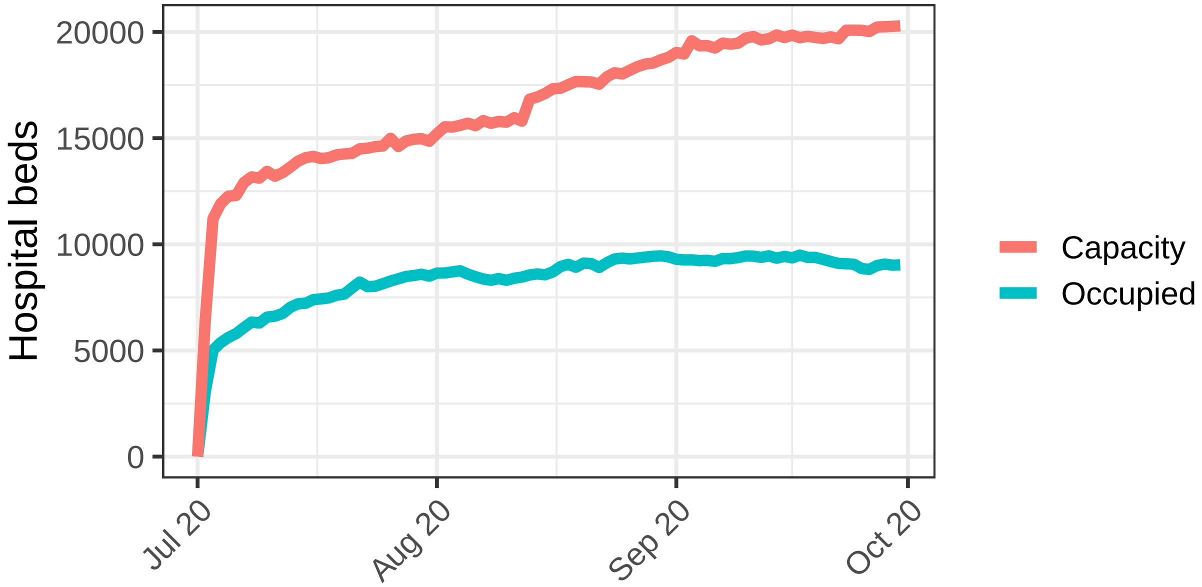

Figure S19: Hospital occupancy and capacity over time.

As capacity has not been exceeded by occupancy, we do not model excess COVID deaths as a consequence of hospital overflow.

D.2 Hospitalisation and fatality rates

Table S5: Age-specific symptomatic-hospitalisation rates (SHR) and hospital-fatality rates (HFR).

	SHR	HFR
**0-4**	0.14	0.07
**5-14**	0.07	0.03
**15-69**	0.16	0.09
**70+**	0.43	0.32

There is underreporting of hospital admissions in the dataset used to estimate hospitalisation and fatality rates for the Philippines. To correct for the missing admissions data, we make some assumptions about which entries were incorrect.

- Define F = fatal outcome (which we have in the dataset); R = recorded admission (which we have in the dataset); and A = admitted (which we don't know, because we know that not all of the admissions were recorded)
- We want to estimate $p(A)$
- Calculate $p(F)$ from the data (e.g. 13.75% for people aged 70+)
- Calculate $p(R)$ from the data (e.g. 12.74% for people aged 70+)
- Calculate $p(F|R)$ from the data (e.g **31.79**% for people aged 70+ – this is the HFR)
- Calculate $p(R|F) = p(F|R) \times p(R)/p(F) = 31.79\times\ 12.74/13.75 = 29.46\%$
- Assume the probability that admission was recorded does not depend on disease outcome:
- $p(R|A) = p(R|F) = 29.46\%$
- Calculate $p(A) = p(R)/p(R|A) =$ **43.26**% – this is the SHR

The key assumptions/approximations are:

1. The death data are correct: $p(F) =$ sCFR
2. The probability that admission was recorded does not depend on disease outcome

3. We model the epidemic as if there were not deaths among people who were not hospitalised

D.3 Other parameters

Table S6: Parameters used in the model

Parameter	Value	Description	Source	Label
Thd	8.00	Expected time to death in hospital	Knock et al. (2021)	$T^{H:D}$
Threc	10.00	Expected time to recovery in hospital	Knock et al. (2021)	$T^{H:R}$
red	0.58	Relative reduction in transmission for asymptomatic infectious	Byambasuren et al. (2020)	ϵ
Text	4.60	Incubation period	Knock et al. (2021)	$T^{E:I}$
p1	0.60	Probability to be symptomatic	Knock et al. (2021)	p^{I^s}
Tsh	4.50	Expected time to hospital for symptomatic infection	Knock et al. (2021)	$T^{I^s:H}$
Ts	4.00	Expected time to recovery for symptomatic infection	Knock et al. (2021)	$T^{I^s:R}$
Ta	2.10	Expected time to recovery from asymptomatic infection	Knock et al. (2021)	T^{I^a}
remote_learning_rel_value	0.37	Relative effectiveness of remote education	National Economic and Development Authority (2021)	

E Epidemic model

E.1 Contact rates

Contact matrices define the mean number of contacts per day reported between groups of individuals and are important components of the SEIR model. Denoted by $M(t)$, they feature explicitly in the force of infection (FOI) term, and are based on contact survey data. There exists an extensive literature deriving coefficients for heterogeneous mixing with respect to age and geography. However, few studies estimated contact structures with respect to economic sector. For the purpose of this study, we account for the heterogeneity in contact rates between sectors. We synthesize a contact matrix based on a contact survey conducted in 2012 in France (Béraud et al., 2015). While more recent contact surveys have been conducted, to our knowledge, this is the only survey that includes sector-specific and work-related information of respondents. Contact rates from eight sectors are mapped onto the 34 sectors in the model.

Entry $M_{ij}(t)$ counts the number of contacts per day a person in group i expects to make with people in group j at time t in such a way that exposes group i to infection from group j. Here, i and j index the N working sectors and the four non-working groups, and the contact matrix is created by adding different types of contacts together. We account for a reduction in transmission risk due to NPIs or working from home via a reduction in effective contact rates, which change over time, and where each day t belongs to a period τ that has a constant economic configuration across its duration. Furloughed and unemployed workers are considered as non-working.

We construct contact matrix $M(t)$ as the sum of four matrices: $M^{\text{com}}(t)$ (community contacts), $M^{\text{WW}}(t)$ (worker-to-worker contacts), $M^{\text{CW}}(t)$ (consumer-to-worker contacts) and $M^{\text{WC}}(t)$ (worker-to-consumer contacts) . Opening of sectors, including hospitality and education, increases transmissions via contact rates in the matrices. Refining the matrices $M^{\text{com}}(t)$, $M^{\text{WW}}(t)$, $M^{\text{CW}}(t)$ and $M^{\text{WC}}(t)$ in this study is fundamental to estimating DAEDALUS.

We use values extracted from the survey published with (Béraud et al., 2015) as follows:

Community contacts

- Any contact made at home, in a vehicle or other private place, retail outlet, public transport, leisure facilities, with loved ones in a closed place ("Chez des proches en

lieux clos"), open place (park, street)

- Disaggregated by age group (0 – 4; 5 – 19; 20 – 64; 65+)

Worker–worker contacts

- Contacts made at work (office, studio, etc.) and which are reported to be made (almost) every day, or a few times per week
- Disaggregated by sector
- Individuals who stated that they are in employment
- Individuals who are of working age (20 – 64)

Consumer–worker

- Contacts made at work (office, studio, etc.) and which are reported to be a few times per month, a few times per year or less often, for the first time
- Disaggregated by sector
- Individuals who stated that they are in employment
- Individuals who are of working age (20 – 64)
- If more than 20 contacts are made by the individual, the survey respondent could state the total number of contacts made instead of listing all individual contacts. If this was the case, this number was used instead of the sum of individual contacts made

E.1.1 Matrix $M^{\mathbf{com}}(t)$

Community contacts (represented in matrix $M^{\text{com}}(t)$) are any contacts made that are unrelated to the workplace. This includes contacts in the household, during travel to and from the workplace and non-work-related travel, outside spaces, school, leisure activities (e.g. meeting friends), retail outlets (e.g. supermarkets), and contacts made in the hospitality or service sectors. When sectors are partially or fully opened, we account for additional transmission risk from contacts between consumers in matrix $M^{\text{com}}(t)$. As such, in addition to household contacts, contacts are being made when consuming products or services from specific sectors. Opening the hospitality sector, for example, will increase the community transmission as consumers meet in pubs and restaurants. The contact rates show the average contact rate for the community. The columns of the community matrix $M^{\text{com}}(t)$ are weighted by the size of the workforce (measured in headcounts) in each sector. The values of row sums depends on the extent to which given sectors are open.

We write matrix $M^{\text{com}}(t)$ as a sum of its constituent parts, representing intra- and

inter-household interactions (L), school interactions (S), hospitality interactions (H) and travel interactions (T):

$$M^{\text{com}}(t) = M^{(L)} + M^{(S)}(t) + M^{(H)}(t) + M^{(T)}(t)$$

Matrix $M^{(L)}$ is estimated using as a basis the contact matrix for "all locations" from (Prem et al., 2017). This is a 16-by-16 matrix ($M^{(0)}$), for five-year age bands a up to age group 75+. We map it to a four-by-four matrix $M^{(1)}$ corresponding to the four age groups g used in the DAEDALUS model, using population sizes, $\hat{P}_a$:

$$M^{(1)}_{gg'} = k \frac{\sum_{a \in g} \hat{P}_a \sum_{a' \in g'} M^{(0)}_{a,a'}}{\sum_{a \in g} \hat{P}_a}.$$

Using P_g to represent the population sizes of the DAEDALUS age groups,

$$P_g = \sum_{a \in g} \hat{P}_a,$$

a single scalar k is found so that the average number of contacts across all age groups is 3.4, in order to match the average contacts per person in Béraud et al. (2015):

$$\frac{\sum_g P_g \sum_{g'} M^{(1)}_{gg'}}{\sum_g P_g} = 3.4.$$

Contacts from all groups j to working groups i depend on the age group of the group ($g(i)$), and the fraction of the age-population represented in group i, where w^*_j is the maximum number of people in group j:

$$M^{(L)}_{ij} = M^{(1)}_{g(i),g(j)} \frac{w^*_j}{P_{g(j)}}$$

for i and j including all groups (working and non-working). Each group i contains people that belong to only one age group g. We refer to the age group of the people in group i as $g(i)$. Then $P_{g(j)}$ is the number of people in the age group of group j, so $P_{g(j)} = w^*_j$ for age groups 0 to 4, 5 to 14 and 70+, and $P_{g(j)} = \sum_{j \in \{1,\ldots,N,N+4\}} w^*_j$ for ages 15 to 69.

Students are 'consumers' of education services. In the education sector, we account for the number of contacts between students going to school or university. School contacts are estimated separately in two age groups (pre-school age: 0 – 4; school age: 5 – 14). Diagonal matrix $M^{(S)}(t)$ counts the contacts in schools. It has entries of zero for groups g not in school, and a value of 1.66 for g=0 to 4 years old, and a value of 7.34 for g=5 to 14 year

olds, and we write $M^{(S0)} = \{1.66, 7.34, 0, 0\}$. Then

$$M_{ii}^{(S)}(t) = x_{S,\tau}^2 A_{g(i)}^{(S0)}. \tag{10}$$

The value $x_{S,\tau}$ is the extent to which schools are open in the period τ, where $t \in \tau$, so that the number of contacts per person scales linearly according to closure.

Matrix $M^{(H)}(t)$ gives the contacts made in the hospitality sector. Each age group makes an average of 0, 0.5, 1 and 1.5 total contacts for age groups 0-4, 5-14, 15-69, and 70+, respectively Béraud et al. (2015). These contacts are made in proportion to population squared, so we can write

$$M_{ij}^{(H)}(t) = x_{H,\tau}^2 \frac{M_{g(i)}^{(H0)} w_j^*}{\sum_{j'} w_{j'}^*} \tag{11}$$

with $M^{(H0)} = \{0, 0.5, 1, 1.5\}$, and $M_{ij}^{(H)} = 0$ for $g(i) \neq g(j)$.

The value $x_{H,\tau}$ is the workforce-weighted average extent to which the hospitality sectors are open in the period τ, so that the number of contacts per person scales linearly according to closure:

$$x_{H,\tau} = \frac{\sum_i x_{i\tau} w_i^*}{\sum_i w_i^*}$$

where we sum over only the hospitality sectors; for the Philippines, $i \in \{22\}$: sector "hotels and restaurants".

Matrix $M^{(T)}(t)$ counts contacts between working people, representing travel. We assume that transport contacts only add to the infection risk if the sector is open and the workers travel to and from their workplace. It is assumed that there are $M^{(T0)}$ contacts per person working with all other people, but we only count those with other working groups (so that each person has less than $M^{(T0)}$ contacts on average), and share the contacts out proportionally among them:

$$M_{ij}^{(T)}(t) = w_{j\tau}(1 - p_{i\tau})(1 - p_{j\tau}) \frac{M^{(T0)} w_j^*}{\sum_g P_g} \tag{12}$$

for $i = 1, ..., N$. $M_{ij}^{(T)} = 0$ for $i > N$.

$p_{i\tau}$ is the proportion of workers from sector i working from home during period τ, and $(1 - p_{i\tau})(1 - p_{j\tau})$ scales contacts between workers superlinearly to approximate the reduced transmission between commuting workers: there should be fewer contacts per person on average, and there should be fewer people having these contacts. We reduce the transmission rates within the groups as a proxy for moving the individuals out of the group.

Also in this equation, $x_{j\tau}$ scales the numbers of contacts linearly with respect to sector closure. At the same time, the number of people in the compartments will be reduced by their sector closure, $x_{i\tau}$. This, in combination with the scaled contacts, leads to superlinear scaling.

E.1.2 Matrix $M^{\mathbf{WW}}(t)$

Worker-to-worker contacts (matrix $M^{\mathrm{WW}}(t)$) describe the at-work contacts in sectors, i.e. the number of contacts per day reported by an individual actively working in the same sector. We use values from (Béraud et al., 2015) corresponding to contacts recorded to have happened at work and frequently (reported as a contact made almost every day). At work contacts at low frequency are classified as worker-consumer contacts. We scale these values to match expected worker-worker contacts from \citet{Prem}, and we denote them b_i (values given per sector in Table S4).

Matrix $M^{\mathrm{WW}}(t)$ is diagonal owing to lack of data regarding between-sector contacts.

$$M_{ii}^{\mathrm{WW}}(t) = w_{i\tau}(1 - p_{i\tau})^2 b_i, \tag{13}$$

for the $i = 1, ..., N$ working groups, with the number of contacts adjusted according to at-home working ($p_{i\tau}$) and sector workforce present ($w_{i\tau}$). The workforce present is related to sector openness via the production function, $w_{i\tau} = x_{i\tau}^{1/\alpha}$. Note that $b_i = 0$ for $i > N$. As before, there is superlinear scaling of contacts with respect to working from home. There is linear scaling with respect to sector closure: that is, there are fewer contacts per person, but we do not approximate there being fewer people having them. This is because the latter is accounted for in the movement of people out of the group upon its closure.

E.1.3 Matrix $M^{\mathbf{CW}}(t)$

Consumer-to-worker contacts (matrix $M^{\mathrm{CW}}(t)$) describe contacts experienced by workers from consumers per sector, denoted c_i. From the (Béraud et al., 2015) survey, contacts experienced by workers from consumers are defined by those contacts recorded to have happened at work less frequently than every day (i.e. recorded as a few times a week, a few times a month, a few times a year or less often, or for the first time). These absolute values are scaled so that average number of contacts weighted by sector size matches the expected number of contacts with non-workers by age according to (Prem et al., 2017).

Then

$$M_{ij}^{\text{CW}}(t) = x_{i\tau}(1 - p_{i\tau})\frac{c_i w_j^*}{\sum_{j'}^{N+4} w_{j'}^*}, \tag{14}$$

for $j = 1, ..., N+4$. $c_i = 0$ for $i > N$.

Here, there is linear scaling of $M_{ij}^{\text{CW}}(t)$ with respect to working from home, and linear scaling with respect to sector closure, which becomes superlinear scaling for sectors as individuals are moved out of the compartment, as with matrix $M^{\text{WW}}(t)$.

E.1.4 Matrix $M^{\mathbf{WC}}(t)$

Worker-to-consumer contacts (matrix $M^{\text{WC}}(t)$) describe contacts experienced by consumers from workers per sector, denoted d_i. These values are reciprocal to those in matrix $M^{\text{CW}}(t)$: if there are M_{ij}^{CW} contacts from group j to a person in group i, and there are w_i^* (w_j^*) people in group i (j), then $M_{ji}^{\text{WC}} = M_{ij}^{\text{CW}} w_i^* / w_j^*$.

E.2 State transitions

Possible transitions between disease states are shown in Figure S20. Transition rates are functions of time t and group identity j (where the groups are the 34 sectors and the four age groups).

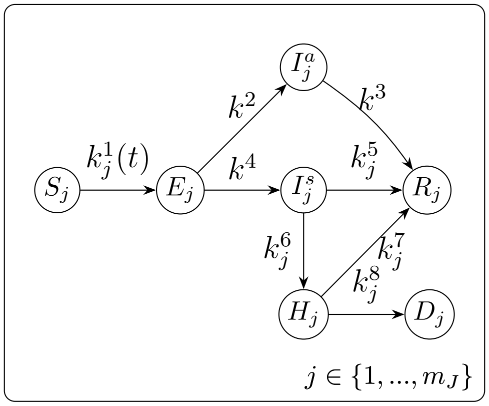

Figure S20: Disease state transitions. S: susceptible. E: exposed. I^a: asymptomatic infectious. I^s: symptomatic infectious. H: hospitalised. R: recovered. D: died. j: stratum. m_J: number of strata.

E.3 Transition rates

E.3.1 Rate of infection

The rate of infection of susceptible individuals, $k^1(t)$, is defined as

$$k^1(t) = \rho(t)\beta\left(M(t) \cdot I^{(eff)}(t)/N\right), \tag{15}$$

which uses the effective number of infectious people,

$$I^{(eff)}(t) = \epsilon I^a(t) + I^s(t). \tag{16}$$

In Equations (15) to (16), $\rho(t)$ is the time-dependent modifier of the rate of infection, β; $M(t)$ is the 38-by-38 contact matrix between groups; ϵ is the infectiousness of asymptomatic relative to symptomatic individuals, and $I^{\cdot}$ is the vector of number of infectious asymptomatic (I^a) and symptomatic (I^s).

E.3.2 Other rates

$$k^2 = (1 - p^{I^s})/T^{E:I}$$

is the rate to asymptomatic infectiousness, where p^{I^s} is the probability to become symptomatic, and $T^{E:I}$ is the expected duration of the latent period before the onset of infectiousness;

$$k^3 = 1/T^{I^a}$$

is the rate of recovery from asymptomatic infection;

$$k^4 = p^{I^s}/T^{E:I};$$

is the rate of symptom onset;

$$k^5 = (1 - p^H)/T^{I^s}$$

is the rate of recovery from symptomatic infection, where p^H is the probability to be hospitalised, and $T^{I^s} = p^H T^{I^s:H} + (1 - p^H)T^{I^s:R}$ is the expected time to be in compartment I^s: $T^{I^s:H}$ is the expected duration before hospitalisation and $T^{I^s:R}$ is the expected duration before recovery.

Then

$$k^6 = p^H/T^{I^s}$$

is the rate of hospitalisation following symptomatic infection.

$$k^7 = (1 - p^D)/T^H$$

is the rate of recovery of hospitalised patients, where p^D is the probability to die given hospitalisation, and $T^H = p^D T^{H:D} + (1-p^D)T^{H:R}$ is the expected time to be in compartment H: $T^{H:D}$ is the expected duration before death and $T^{H:R}$ is the expected duration before recovery.

Finally,

$$k^8 = p^D/T^H$$

is the rate of death following hospitalisation.

F Fitted parameters

Table S7: Parameters learnt in fitting model to data

Parameter	Value	Description
seed	-54.44	Time of epidemic seeding
R0	2.63	Basic reproduction number
	0.6437	Transmission modifier (March)
	0.4787	Transmission modifier (April)
	0.3489	Transmission modifier (May)
	0.7279	Transmission modifier (June)
	0.5093	Transmission modifier (July)
	0.5260	Transmission modifier (August)
	0.5117	Transmission modifier (September)
